# HpARI Protein Secreted by a Helminth Parasite Suppresses Interleukin-33

**DOI:** 10.1016/j.immuni.2017.09.015

**Published:** 2017-10-17

**Authors:** Megan Osbourn, Dinesh C. Soares, Francesco Vacca, E. Suzanne Cohen, Ian C. Scott, William F. Gregory, Danielle J. Smyth, Matilda Toivakka, Andrea M. Kemter, Thierry le Bihan, Martin Wear, Dennis Hoving, Kara J. Filbey, James P. Hewitson, Holly Henderson, Andrea Gonzàlez-Cìscar, Claire Errington, Sonja Vermeren, Anne L. Astier, William A. Wallace, Jürgen Schwarze, Alasdair C. Ivens, Rick M. Maizels, Henry J. McSorley

**Affiliations:** 1 MRC Centre for Inflammation Research, University of Edinburgh, Queen’s Medical Research Institute, 47 Little France Crescent, Edinburgh EH16 4TJ, UK; 2 Department of Respiratory, Inflammation and Autoimmunity, MedImmune Ltd, Granta Park, Cambridge CB21 6GH, UK; 3 Institute of Immunology and Infection Research, and Centre for Immunity, Infection and Evolution, School of Biological Sciences, Ashworth Laboratories, University of Edinburgh, West Mains Road, Edinburgh EH9 3JT, UK; 4 Wellcome Centre for Molecular Parasitology, Institute for Infection, Immunity and Inflammation, University of Glasgow, Sir Graeme Davies Building, 120 University Place, Glasgow G12 8TA, UK; 5 SynthSys, The Kings Buildings, University of Edinburgh, Edinburgh EH9 3BF, UK; 6 The Edinburgh Protein Production Facility (EPPF), Wellcome Trust Centre for Cell Biology (WTCCB), University of Edinburgh, King's Buildings, Max Born Crescent, Mayfield Road, Edinburgh EH9 3BF, UK; 7 Centre for Immunology and Infection, Department of Biology, University of York YO10 5DD, York, UK; 8 Department of Pathology, Laboratory Medicine, Royal Infirmary of Edinburgh, Edinburgh, UK

**Keywords:** IL-33, helminth, parasite, allergy, asthma, immunomodulation

## Abstract

Infection by helminth parasites is associated with amelioration of allergic reactivity, but mechanistic insights into this association are lacking. Products secreted by the mouse parasite *Heligmosomoides polygyrus* suppress type 2 (allergic) immune responses through interference in the interleukin-33 (IL-33) pathway. Here, we identified *H. polygyrus* Alarmin Release Inhibitor (HpARI), an IL-33-suppressive 26-kDa protein, containing three predicted complement control protein (CCP) modules. *In vivo*, recombinant HpARI abrogated IL-33, group 2 innate lymphoid cell (ILC2) and eosinophilic responses to *Alternaria* allergen administration, and diminished eosinophilic responses to *Nippostrongylus brasiliensis*, increasing parasite burden. HpARI bound directly to both mouse and human IL-33 (in the cytokine’s activated state) and also to nuclear DNA via its N-terminal CCP module pair (CCP1/2), tethering active IL-33 within necrotic cells, preventing its release, and forestalling initiation of type 2 allergic responses. Thus, HpARI employs a novel molecular strategy to suppress type 2 immunity in both infection and allergy.

## Highlights

• HpARI is a suppressor of IL-33 release and consequent allergic sensitization
• HpARI binds active IL-33 and nuclear DNA, tethering IL-33 within necrotic cells
• HpARI is active against both human and murine IL-33

## Graphical Abstract

**Figure:**
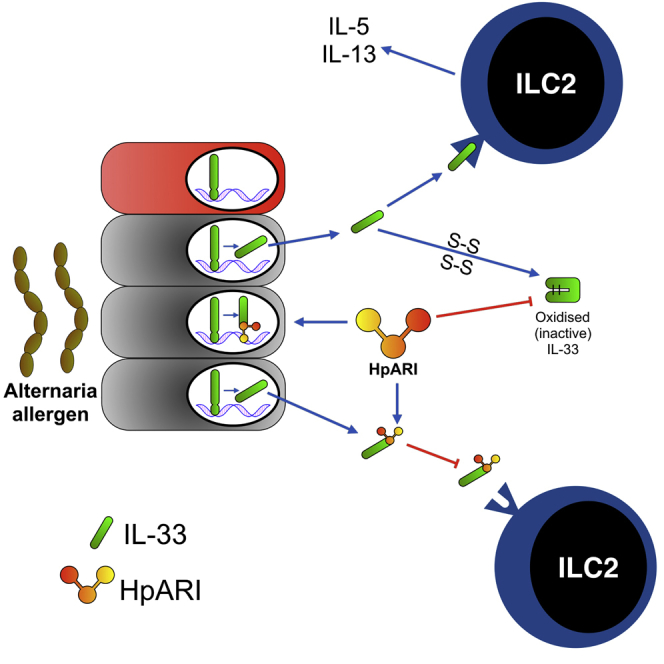

## Teaser

Osbourn et al identified HpARI, a protein secreted by a helminth parasite that is capable of suppressing allergic responses. HpARI binds to IL-33 (a critical inducer of allergy) and nuclear DNA, preventing the release of IL-33 from necrotic epithelial cells.

## Introduction

Infection with helminth parasites negatively correlates with prevalence of allergic disease, and parasitic infection is associated with immunosuppression (Maizels and McSorley, 2016). Many researchers, ourselves included, have demonstrated that helminths release immunomodulatory proteins to control anti-parasite immune responses and maintain their persistence in the host (Maizels and McSorley, 2016). We previously showed that the excretory–secretory products of the mouse intestinal parasite *Heligmosomoides polygyrus* (HES) suppress allergic responses in mouse models of asthma (Buck et al., 2014, McSorley et al., 2015, McSorley et al., 2014, McSorley et al., 2012). HES administration blocks the interleukin-33 (IL-33) response to inhaled *Alternaria* (fungal) allergen (McSorley et al., 2014) leading to reduced type 2 innate lymphoid cell (ILC2) responses and abrogating lung pathology.

*IL33* and its receptor (*IL1RL1*) are both among the 10 genes most strongly linked to allergic sensitization (Bønnelykke et al., 2013) and asthma (Bønnelykke et al., 2014, Moffatt et al., 2010) in genome-wide association studies. IL-33 concentration is increased in the lungs of severe asthmatics (Castanhinha et al., 2015, Saglani et al., 2013), correlating negatively with lung function (Christianson et al., 2015). Respiratory viral infections are implicated in both initiation and exacerbation of asthma, an effect that is also associated with IL-33 release (Jackson et al., 2014, Saravia et al., 2015).

The IL-33 receptor (ST2, IL1RL1, IL-33R) is expressed by a wide range of cells, notably T cells, macrophages, endothelial cells, epithelial cells, and ILC2 (Cayrol and Girard, 2014). Through these interactions, IL-33 drives type 2 immune responses in a range of diseases including asthma, atopic dermatitis, food allergy, COPD, eosinophilic inflammatory bowel disease, eosinophilic esophagitis, and age-related macular degeneration (De Salvo et al., 2016, Liew et al., 2016, Simon et al., 2015, Tordesillas et al., 2014). IL-33 is a member of the IL-1 family of cytokines. It is stored preformed in the nucleus bound to heterochromatin, and its dominant function is as an alarmin cytokine. Active IL-33 is released from the nucleus under conditions of necrosis, while during apoptosis active caspases cleave IL-33 within its receptor-binding domain, abolishing activity (Lefrançais and Cayrol, 2012). Although the full-length, 30 kDa form of IL-33 is functional, the activity of IL-33 is increased 10-fold through cleavage between the DNA-binding and receptor-binding domains by proteases such as calpain-2 (Hristova et al., 2016), neutrophil elastase, cathepsin G (Lefrançais et al., 2012), and mast cell tryptase (Lefrançais et al., 2014) releasing 18–21 kDa mature forms. Active IL-33 is released in a reduced form, which under physiological conditions rapidly oxidizes, forming new disulfide bonds and changing conformation, rendering it unable to bind to the IL-33R beyond a short temporal and spatial range (Cohen et al., 2015).

Here, we identified *H. polygyrus* Alarmin Release Inhibitor (HpARI), a HES-derived recombinant protein that can replicate the IL-33-suppressive effects of total HES. HpARI bound directly to active murine or human IL-33 and nuclear DNA. This dual binding blocked the interaction of IL-33 with its receptor, and tethered IL-33 within necrotic cells, preventing its release, and blocking allergic response initiation. Thus, HpARI prevents initiation of parasite-toxic IL-33-mediated type 2 immune responses and suppresses the development of allergic airway inflammation.

## Results

### In Vitro Suppression of IL-33 by HES

**Figure 1:**
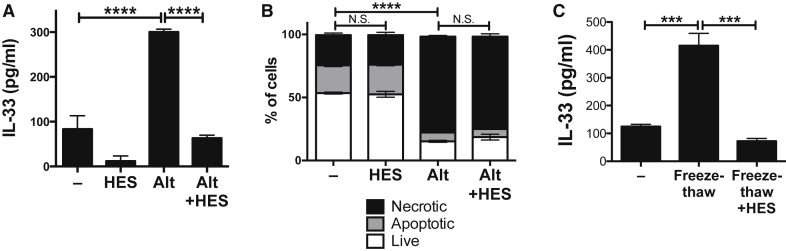
HES Suppression of IL-33 (A) IL-33 levels (ELISA) in supernatants of naive murine lung cells (1 × 10^5^ per well), cultured for 1 hr with *Alternaria* (Alt) allergen (200 μg/ml) and HES (10 μg/ml). (B) Propidium iodide (PI) and annexin V (AnnV) staining of cells from (A) was used to assess apoptosis (PI–AnnV+) versus necrosis (PI+AnnV+). (C) IL-33 levels (ELISA) in supernatants of naive murine lung cells, freeze-thawed in the presence of HES. All data shows SEM of 2–3 replicates, and are representative of 2–3 repeat experiments. Error bars show SEM.

Previous studies established that HES ablates detectable IL-33 in the bronchoalveolar milieu after *Alternaria* allergen administration, suppressing downstream allergic responses (McSorley et al., 2014). To further investigate the IL-33-suppressive activity of HES, we developed an *in vitro* assay for IL-33 release: a single cell suspension of naïve total murine lung cells cultured for 1 hr in the presence of *Alternaria* allergen and HES. In this assay, HES markedly reduced the amount of IL-33 in culture supernatants, as detected by ELISA (Figure 1A).

IL-33 is released from lung epithelial cells under conditions of necrosis, whereas activated caspases cleave IL-33 within the IL-1-like cytokine domain, inactivating IL-33 under conditions of apoptosis (Lefrançais and Cayrol, 2012). We therefore hypothesized that HES could be activating caspase and/or apoptosis pathways. Propidium iodide and annexin V staining showed that cells incubated with *Alternaria* allergen were highly necrotic and that this was unaffected by the presence of HES (Figure 1B). Necrosis induced by freeze-thaw treatment of lung cells also resulted in substantial IL-33 release, which again was abrogated by treatment of cells with HES immediately prior to freezing (Figure 1C). Therefore we conclude that HES suppression of IL-33 does not depend on activation of the apoptosis pathway, but instead acts on pre-formed IL-33 released from necrotic cells.

### Identification and Characterization of HpARI Protein

**Figure 2:**
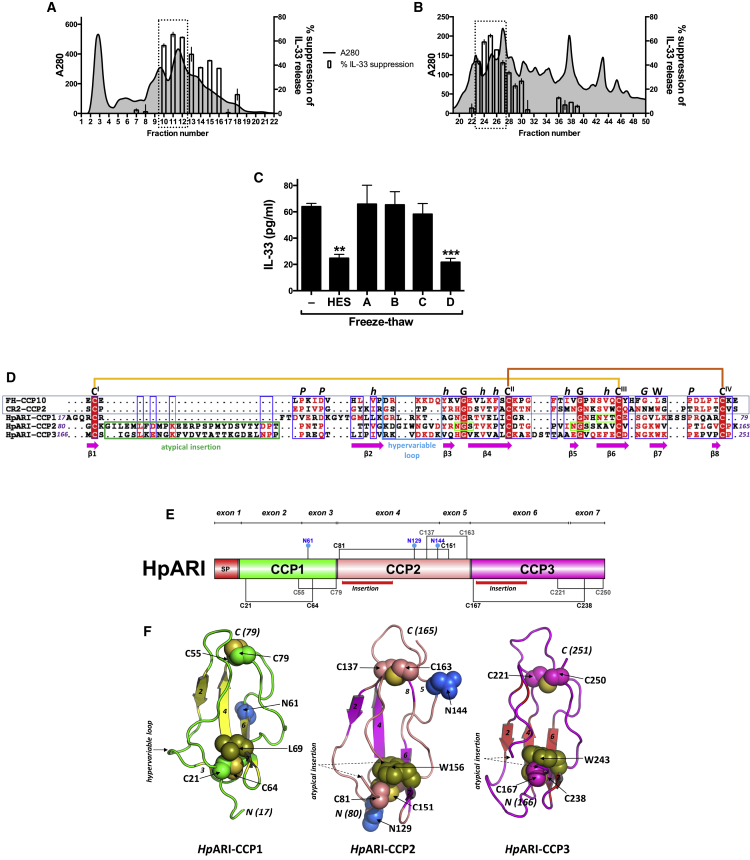
Identification and Bioinformatic Characterization of HpARI Sequence and Structure (A) IL-33 suppression by HES size fractions. (B) IL-33 suppression by HES charge fractions. Data in (A) and (B) are percentage suppression of the IL-33 signal compared to *Alternaria*-only control. Dotted rectangles indicate peaks used for selection of candidates. (C) IL-33 levels (ELISA) in supernatants of naïve murine lung cells, freeze-thawed in the presence of supernatants of HEK293T cells transfected with four candidate genes. Mean and SEM are shown of three replicate wells, representative of three repeat experiments. (D) Alignment of HpARI CCP-like modules with complement receptor type 2 CCP2 (CR2-CCP2) and complement factor H CCP10 (FH-CCP10). The putative disulfide bonding pattern (C^I^-C^III^; C^II^-C^IV^), conserved tryptophan (W) and structurally-important proline (P), glycine (G), and hydrophobic amino acid residues (h), characteristic of a CCP-module are indicated. Atypical insertions in CCP2/3 (green box), the hypervariable loop (cyan box), and beta-strands (pink arrows) are indicated, based on known CCP secondary structure of CR2-CCP2, as well as three potential N-linked glycosylation sites (light green box). (E) HpARI domain schematic, with putative disulfide bonding pattern and location of insertions indicated. (F) Structural models of the three HpARI CCP-like modules. Error bars show SEM.

A process of fractionation, screening, and proteomic analysis of HES was used to identify candidate IL-33-suppressive proteins. Gel filtration and anion exchange FPLC were used to fractionate HES by size and charge, respectively. IL-33 suppressive activity peaked around size fraction 11 (Figure 2A) and charge fraction 25 (Figure 2B). Each size and charge fraction was subjected to trypsin digestion followed by liquid chromatography-electrospray tandem mass spectrometry (LC-MS/MS), and the exponentially modified protein abundance index (emPAI) value for each HES protein in every fraction was calculated, and compared to the profile of IL-33 suppression.

By size fractionation, 220 proteins were found with emPAI values which peaked around size fraction 11 (peak value in fractions 10–12), while 371 proteins were found with emPAIs which peaked around charge fraction 25 (peak value in fractions 23–27), 54 of which were shared between the two fractionation techniques. Proteins were prioritized wherein more than one peptide was detected in size fraction 11 and charge fraction 25, resulting in a short-list of 25 candidate proteins (Table S1). The emPAI values for each of these 25 candidates for all size and charge fractions was then manually compared to the IL-33 suppression profile (Figure S1A), and 4 candidates were selected for initial screening (Figure S1B).

The 4 candidate IL-33 suppressive genes were transfected into HEK293T cells for expression, and screened for suppression of the IL-33 signal *in vitro*. Of the 4 candidates, only the transcript named Hp_I08176_IG02172_L1157 in our in-house sequencing (candidate “D” in Figure 2C) significantly suppressed IL-33; this protein was consequently renamed as *H. polygyrus* Alarmin Release Inhibitor (HpARI). Subsequently, an identical transcript was found at WormBase Parasite: HPBE_0000813301.

The *HpARI* gene is made up of 7 exons, encoding a 251-aa protein including a 16-aa signal peptide motif (Figure S2A), with a deduced mature molecular weight of 26 kDa. The mature protein contains three predicted Complement Control Protein (CCP)-like modules (also known as Short Consensus Repeats (SCRs) or sushi-domains, PFAM00084) (Figure 2D). CCP1–3 all contain features of a CCP module such as the four consensus Cysteine residues (Cys^I^ to Cys^IV^, consistent with formation of disulfide bonds in a Cys^I^-Cys^III^ and Cys^II^-Cys^IV^ pattern), the Trp/Leu residue between Cys^III^ and Cys^IV^ and other structurally important residues typical of a CCP module (Figures 2D and 2E and STAR Methods) (Kirkitadze and Barlow, 2001, Soares et al., 2005). Compared to archetypal CCP modules (Soares and Barlow, 2005), all three are atypical in part with divergent sequence features, including an absence of conserved Proline residues after Cys^I^ in CCP1, and atypical insertions of ∼20 amino acid residues between Cys^I^ and Cys^II^ in CCP 2 and CCP3, which are unique compared to previously identified CCP domains. Each CCP module is encoded by two exons with the second exon boundary in each case falling between adjacent predicted CCP modules (i.e. between Cys^IV^ of one module and Cys^I^ of the next) lending further support to the discerned domain boundaries (Figure 2E and Figure S2A).

The three predicted HpARI CCP module sequences were modelled individually based upon their top ranked CCP module template structures. Each CCP module 3-D model is characterized by a β-sheet framework, held together by two disulfide bridges. Other key structural features such as the location of the buried Trp/Leu, hypervariable loop, and potential N-glycosylation sites are indicated along with the relative positions of the novel insertions in CCP2 and CCP3, which could not be modelled on conventional experimentally determined CCP module structures (Figure 2F).

### In Vitro and In Vivo IL-33 Suppression by HpARI

**Figure 3:**
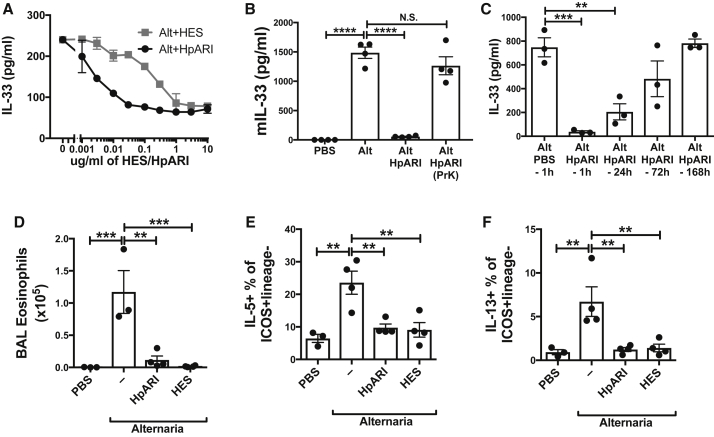
HpARI Suppresses Responses to *Alternaria* Allergen (A) IL-33 levels (ELISA) in supernatants of naive mouse lung cells, cultured for 1 hr in the presence of *Alternaria* (200 μg/ml) and HES or HpARI. (B) IL-33 levels (ELISA) in BAL 1 hr after *Alternaria* allergen administration with HpARI (5 μg) or proteinase K-degraded and heat-treated HpARI (“HpARI (prK)”). (C) IL-33 levels (ELISA) in BAL 1 hr after *Alternaria* allergen administration, with HpARI (5 μg) administered 1, 24, 72, or 168 hr prior to *Alternaria* allergen. (D) BAL eosinophil numbers 24 hr after *Alternaria* allergen, HpARI, and HES administration. (E) Lung ILC2 IL-5 staining from mice in (D). (F) Lung ILC2 IL-13 staining from mice in (D). All data representative of 2–3 repeat experiments, each with 3–4 replicates/mice per group. Error bars show SEM.

Recombinant mature 6-His and Myc-tagged HpARI protein was purified by metal chelating chromatography (Figure S2B), and tested for IL-33 suppression *in vitro*. HpARI was active at <10 ng/ml, while HES required an approximately 50-fold higher concentration for a similar effect (Figure 3A). The IL-33-suppressive activity of HpARI in response to *Alternaria* culture or freeze-thaw was ablated on heat-treatment, as with HES (Figures S3A and S3B).

HpARI also effectively suppressed IL-33 detected in bronchoalveolar lavage (BAL) fluids in response to *Alternaria* allergen *in vivo* (Figure 3B). Again this effect replicated that of HES (McSorley et al., 2014) and suppression was ablated when HpARI was proteolytically cleaved and heat-treated, ruling out a role for non-protein contaminants. In addition, the IL-33-suppressive effects of HpARI could pre-condition airway tissues, substantially reducing the IL-33 response to *Alternaria* allergen 24 hr later, with a degree of protection in some animals even after 72 hr (Figure 3C). Thus, HpARI appears to be a critical IL-33-suppressive factor in HES.

### Suppression of In Vivo Type 2 Responses by HpARI

*Alternaria* exposure induces a rapid T cell-independent eosinophilia within 24 hr of administration. This response is driven by ILC2 cytokine release, and is critically dependent on IL-33 (Bartemes et al., 2012). Recombinant HpARI co-administration with *Alternaria* allergen abrogated BAL eosinophilia (Figure 3D) and lung ILC2 IL-5 (Figure 3E) and IL-13 production (Figure 3F), 24 hr later, again replicating the effects observed with total HES. IL-13-eGFP reporter mice were used to assess ILC2 cytokine responses in the absence of PMA and Ionomycin stimulation, confirming profound suppression of IL-13 reporter expression in ICOS^+^CD90.2^+^IL-33R^+^CD127^+^CD45^+^lineage^–^ ILC2s by HpARI (Figures S3C–S3E).

**Figure 4:**
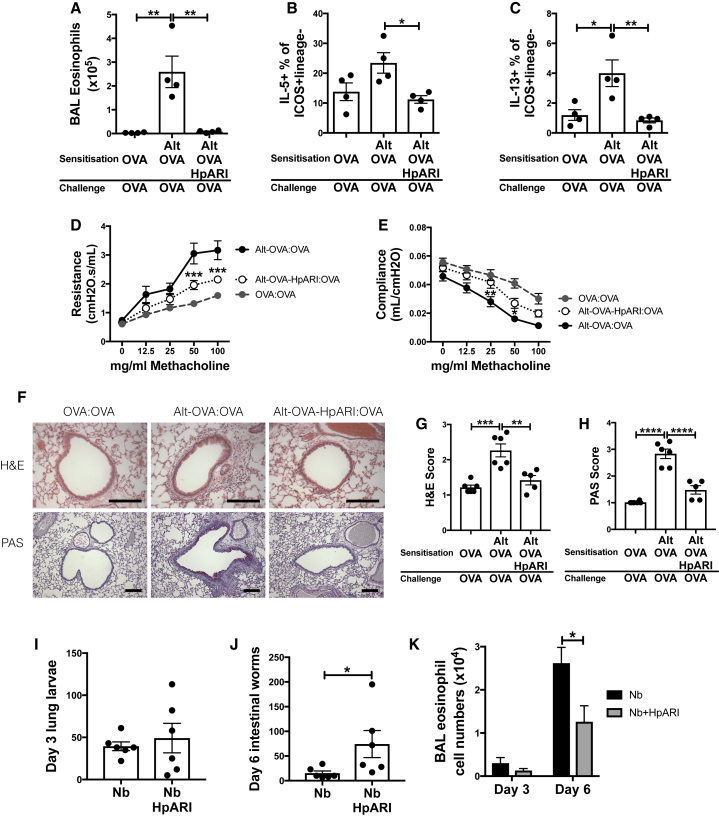
HpARI Suppresses Responses to *Alternaria* Allergen (A) Day 17 BAL eosinophil numbers after *Alternaria* allergen, OVA protein, and HpARI administration on day 0 (sensitization), and OVA protein alone on days 14, 15, and 16 (challenge). (B) Lung ILC2 IL-5 production from mice in (A). (C) Lung ILC2 IL-13 production from mice in (A). (D) Lung resistance in methacholine challenge from mice treated as in (A). (E) Lung compliance in methacholine challenge from mice treated as in (A). (F) H&E- (top panels) and PAS-stained (bottom panels) lung sections from mice treated as in (A). Scale bars indicate 100 μm. (G) H&E scoring of sections from mice treated as in (A). (H) PAS scoring of sections from mice treated as in (A). Alternaria model data representative of 2–3 repeat experiments, each with 4–6 mice per group. (I) Mice were subcutaneously infected with *N. brasiliensis*, and HpARI administered intranasally on days 0, 1, and 2 of infection. Lung larvae were counted 3 days after infection. (J) Day 6 intestinal *N. brasiliensis* worms from mice treated as in (I). (K) Day 3 and day 6 BAL eosinophil numbers from mice treated as in (I). Error bars show SEM.

HpARI was administered in a T cell-dependent model of asthma, in which OVA protein is first co-administered with *Alternaria*, and antigen-specific type 2 responses recalled 2 weeks later by challenge with OVA protein alone (McSorley et al., 2014). Again HpARI replicated the suppressive effects of HES on BAL eosinophilia and lung ILC2 responses (Figures 4A–4C). Furthermore, this suppression led to significantly abrogated lung resistance and compliance at challenge (Figures 4D and 4E), as well as reduced inflammation and mucus production assessed by histological staining (Figure 4F–4H).

Finally, the role of HpARI in parasite infections was addressed using *Nippostrongylus brasiliensis* infection, a parasite which (unlike *H. polygyrus*) migrates through the lung and leads to early IL-33-dependent type 2 responses (Hung et al., 2013). Similarly to the phenotype seen in an IL-33-deficient mouse, HpARI administration did not affect worm burden at early timepoints, but increased numbers of adult parasites found in the intestinal lumen at day 6 (Figures 4I and 4J). This suppression of parasite rejection was associated with reduced BAL eosinophilia, reaching significance at day 6 (Figure 4K). Thus, HpARI abrogates parasite- or allergen-induced IL-33-dependent type 2 immune responses, abrogating parasite ejection and suppressing allergic pathology.

### HpARI Binding to IL-33

**Figure 5:**
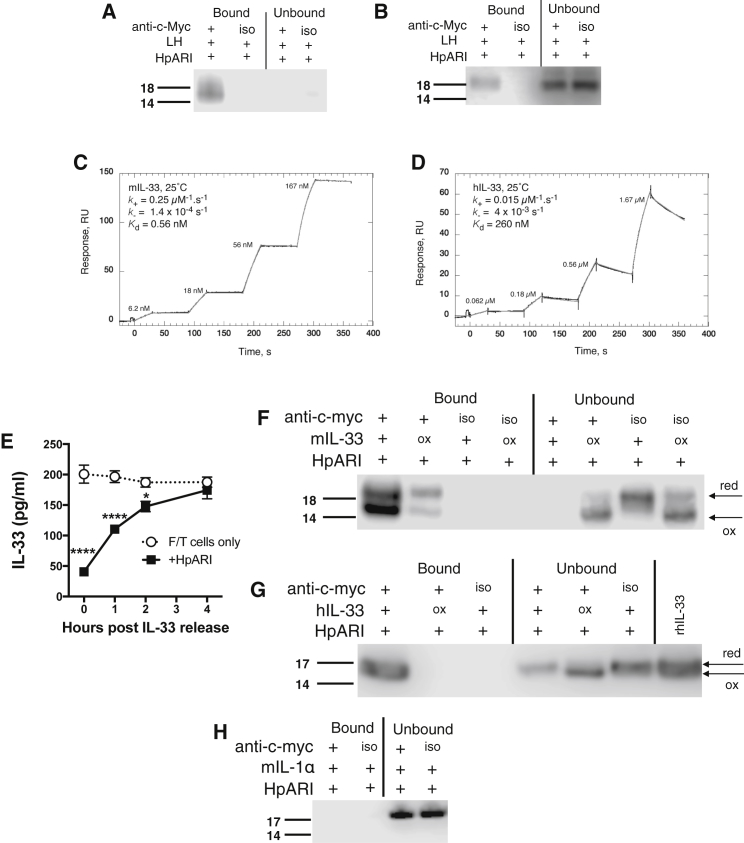
HpARI Binds Active Murine and Human IL-33 (A) Murine IL-33 western blot (non-reducing) of HpARI immunoprecipitation of mouse lung homogenates, using anti-c-Myc antibody, or MOPC isotype control (iso). (B) Human IL-33 western blot (non-reducing) of HpARI immunoprecipitation of human lung homogenates, as in (A). (C) Characterization of the interaction of mouse IL-33 (mIL-33) with HpARI by surface plasmon resonance (SPR - BIAcore T200). Reference corrected single kinetic titration SPR binding curves (black), and a globally fitted 1:1 kinetic binding model (grey). (D) Characterization by SPR of the interaction of human IL-33 (hIL-33) with HpARI, as in (C). (E) IL-33 levels (ELISA) in supernatants of freeze-thawed murine lung cells, incubated at 37°C for 0, 1, 2, or 4 hr, before addition of 1 μg/ml HpARI, and a further incubation for 1 hr at 37°C. (F) Untreated or oxidized recombinant murine IL-33 immunoprecipitated with HpARI as in (A). (G) Untreated or oxidized recombinant human IL-33 immunoprecipitated with HpARI as in (B). (H) Immunoprecipitation experiments repeated with recombinant murine IL-1α, and probed with anti-murine IL-1α. Arrows indicate specific IL-33 or IL-1α bands, and IL-33 reduced (“red”) or oxidized (“ox”) bands. All data are representative of at least two independent repeats. Error bars show SEM.

We hypothesized that HpARI could act by binding directly to IL-33. To investigate this, we incubated Myc-tagged HpARI with murine lung cell homogenates, and immunoprecipitated with anti-c-Myc antibody bound to protein G-coated beads. HpARI immunoprecipitated a clear band at ∼18 kDa in Myc-tagged complexes eluted from anti-c-Myc-coated, but not isotype control-coated beads, as revealed by anti-IL-33 western blotting (Figure 5A). Unbound material (supernatants from co-immunoprecipitation) showed undetectable or very faint bands for IL-33 under these conditions, reflecting the manner in which immunoprecipitation concentrates ligand sufficiently for detection. No band could be detected for full-length IL-33 (30 kDa) in these experiments (data not shown).

Despite human and murine IL-33 sharing only 52% amino acid identity, we found that human IL-33 also co-immunoprecipitates with HpARI after incubation with human lung homogenates, seen as an ∼18 kDa band corresponding to mature human IL-33 (Figure 5B). In this case, unbound human IL-33 could be detected in supernatants from co-immunoprecipitation or control conditions, also at ∼18 kDa.

To biochemically characterize the binding of human and mouse IL-33 with HpARI, we assessed the interactions between these proteins by surface plasmon resonance (SPR) (Figures 5C and 5D). The equilibrium dissociation constant (KD) of HpARI for murine IL-33 is 0.56 ± 0.1 nM, and 260 ± 13 nM for human IL-33.

### Oxidation of IL-33

Recently, it was shown that IL-33 is released in an active reduced form, which is quickly oxidized (<4 hr after release) and inactivated under physiological conditions (Cohen et al., 2015). Commercially-available IL-33 ELISA kits do not differentiate between the reduced and oxidized forms. Therefore we decided to investigate whether HpARI preferentially bound to reduced or oxidized IL-33.

To attain a source of oxidized and reduced IL-33, we subjected lung cells to freeze and thaw-mediated necrosis, harvested IL-33-containing supernatants immediately post-thaw, and incubated these at 37°C for 1–4 hr to oxidize IL-33 (Cohen et al., 2015). When HpARI was added to supernatants directly post-thaw, or up to 2 hr later, it was able to significantly reduce the IL-33 signal as measured by ELISA, whereas by 4 hr post-thaw, no effect of HpARI could be seen (Figure 5E and Figure S4A). Therefore we hypothesized that HpARI binds only to active (reduced) IL-33.

HpARI co-immunoprecipitation experiments were then repeated with either untreated recombinant murine IL-33 (rmIL-33) or rmIL-33 which had been oxidized by incubation for 24 hr at 37°C in tissue culture medium. Eluted complexes were run on non-reducing SDS-PAGE gels to distinguish reduced and oxidized IL-33 by their differential migration under non-reducing conditions, the more compact oxidized form migrating more rapidly (Cohen et al., 2015). A strong bias for binding of HpARI to the reduced form could be seen, with unbound supernatants containing the oxidized form, while no unbound reduced IL-33 could be detected (Figure 5F).

Co-immunoprecipitation was repeated with recombinant human IL-33 (rhIL-33), either untreated or oxidized under the same conditions as applied to murine IL-33. Similarly to murine IL-33, rhIL-33 could only be bound by HpARI in its reduced, active form, with oxidation of IL-33 abolishing its ability to be co-precipitated (Figure 5G).

Finally, we ensured that the binding of HpARI is specific to IL-33, by binding studies with the closely-related IL-1 family cytokine IL-1α. No binding of HpARI to IL-1α could be detected, either by co-immunoprecipitation (Figure 5H) or by SPR (Figure S4B). Thus, HpARI specifically and with high affinity, binds to the active, reduced form of IL-33.

### HpARI Prevents Binding of Active IL-33 to the IL-33 Receptor

**Figure 6:**
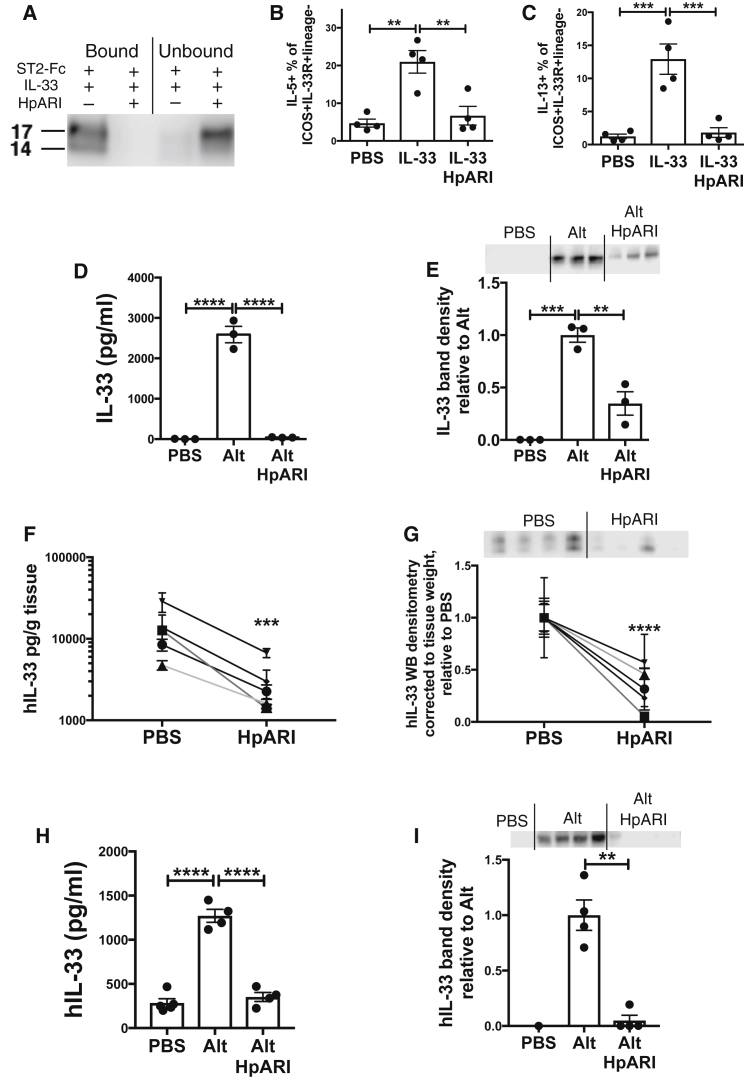
HpARI Blocks IL-33-ST2 Interactions and Inhibits IL-33 Release (A) IL-33 western blot (non-reducing) of ST2-Fc fusion protein immunoprecipitation of recombinant murine IL-33 in the presence or absence of HpARI. (B) Lung ILC2 IL-5 production 24 hr after intranasal administration of recombinant murine IL-33 (200 ng/mouse) with 5 μg HpARI. (C) Lung ILC2 IL-5 production from mice described in (B). (D) Murine IL-33 levels (ELISA) in BAL 15 min after *Alternaria* allergen and HpARI were intranasally administered. (E) Murine IL-33 western blot (∼20 kDa band and densitometry analysis) of BAL from mice described in (D). (F) Human IL-33 levels (ELISA) in supernatants of human lung explants cultured for 1 hr with HpARI. (G) Human IL-33 western blot (∼20 kDa band and densitometry analysis) of supernatants from human lung explants cultures described in (F). (H) Human IL-33 levels (ELISA) in BAL fluid of human IL-33-transgenic mice, 30 min after *Alternaria* allergen and HpARI intranasal administration. (I) Human IL-33 western blot (∼20 kDa band and densitometry analysis) of BAL from human IL-33-transgenic mice described in (H). Mouse data (A–E, H–I) representative of 2–4 repeat experiments, each with 3–4 mice per group. Human data (C and D) shows 5 independent subjects. Error bars show SEM.

To investigate whether HpARI binding IL-33 consequently affected downstream responses to IL-33, we investigated the binding of IL-33 to its receptor ST2. Recombinant mIL-33 was incubated alone or with HpARI, then immunoprecipitation was carried out using an ST2-Fc fusion protein bound to protein G-coated magnetic beads. The presence of HpARI completely blocked immunoprecipitation of rmIL-33 by ST2-Fc (Figure 6A), implying that HpARI prevents IL-33 from binding to its receptor.

Furthermore, when rmIL-33 was administered intranasally to mice, IL-33-mediated ILC2 activation (measured by IL-5 and IL-13 production) was effectively ablated by HpARI co-administration (Figures 6B and 6C). Thus HpARI, through binding to IL-33, can prevent the activation of ILC2s through ST2 ligation.

### HpARI Inhibits Release of IL-33

As HpARI directly binds IL-33, it could also interfere with detection of the cytokine by ELISA through masking epitopes bound by assay antibodies. This could affect our early screening results, (Figures 1, 2, and 3) as these are largely dependent on ELISA to measure concentrations of IL-33. To investigate the possibility of undetectable HpARI-bound IL-33 in BAL supernatants, we measured IL-33 by both ELISA and western blot, as the latter reduces, denatures and dissociates protein complexes. Mice were treated with *Alternaria* allergen and BAL taken 15 min later (at which timepoint the majority of IL-33 released is active and reduced [Cohen et al., 2015]), HpARI coadministration ablated the IL-33 signal by ELISA (Figure 6D), and significantly inhibited (but did not ablate) the IL-33 signal by western blot (Figure 6E), implying that although HpARI binding interferes with IL-33 detection by ELISA, IL-33 release is indeed diminished with HpARI administration. In contrast, HpARI could not affect the release of HMGB1, another nuclear-localised alarmin cytokine released on necrosis, (Figure S4C), demonstrating that the effects of HpARI are specific to IL-33.

To translate these results to human biology, we cultured human lung explants for 1 hr with HpARI, a system and timepoint in which lung explants spontaneously release reduced (active) human IL-33 (Cohen et al., 2015). Similarly to the murine system, a reduction in IL-33 signal was seen with HpARI coadministration, as measured by both ELISA and western blot (Figures 6F and 6G). Furthermore, HpARI was administered with *Alternaria* to human IL-33 transgenic mice (Cohen et al., 2015), where it again suppressed human IL-33 release into the BAL (Figures 6H and 6I). Thus, HpARI reduces the release of both mouse and human IL-33.

### Immunofluorescent Localization of HpARI

To further investigate the mechanism of action of HpARI, we utilized the CMT-64 mouse lung epithelial carcinoma cell line, which we found stores high amounts of IL-33 in the nucleus (Figure S5A). Similarly to lung cells cultured *in vitro*, IL-33 is released from freeze-thawed CMT-64 cells, and this response is suppressed by HpARI (Figure S5B). We then produced an HpARI_mCherry fusion protein, allowing fluorescent localization of HpARI binding, while retaining IL-33-suppressive activity (Figure S5C).

**Figure 7:**
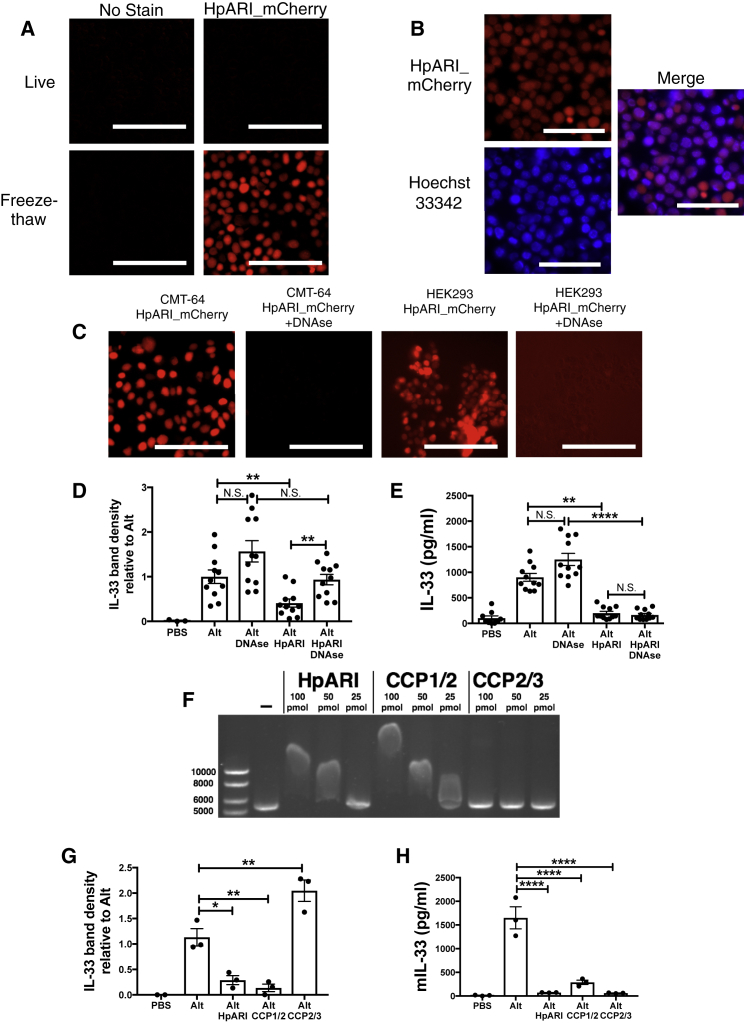
HpARI Binds Nuclear DNA, Tethering IL-33 within Necrotic Cells (A) Live (top panels) or freeze-thawed (bottom panels) CMT-64 cells were incubated for 1 hr at 37°C with 5 μg/ml HpARI_mCherry. (B) HpARI_mCherry-stained freeze-thawed CMT-64 cells, with Hoechst 33342 nuclear co-stain. (C) Freeze-thawed CMT-64 or HEK293T cells were stained with HpARI_mCherry with 100 U/ml DNAse I. (D) Murine IL-33 western blot densitometry of BAL taken 15 min after *Alternaria* allergen, HpARI and DNAse (100 U) intranasal administration. (E) Murine IL-33 levels (ELISA) IL-33 in BAL fluid from mice described in (D) (F) Gel shift assay of linearised plasmid DNA, incubated with 100, 50 or 25 pmol of HpARI, CCP1/2 or CCP2/3 truncated proteins. (G) Murine IL-33 western blot densitometry of BAL taken 15 min after *Alternaria* allergen, HpARI or CCP1/2 or CCP2/3 HpARI truncated proteins intranasal administration. (H) Murine IL-33 levels (ELISA) in BAL from mice described in (G). All data representative of at least 2 repeat experiments. Data in (D) and (E) shows mean and SEM of 3 pooled experiments, data log-transformed for statistical analysis to equalize variances. Scale bars = 100 μm. Error bars show SEM.

Although we found no HpARI_mCherry staining of live CMT-64 cells, binding was evident in freeze-thaw treated necrotic cells (Figure 7A), where it bound in the nucleus (Figure 7B). Surprisingly, we found HpARI_mCherry binds the nucleus independently of IL-33 expression, as similar staining could be seen in HEK293 cells (Figure 7C), from which no IL-33 could be detected (data not shown). As binding of HpARI in the nucleus of CMT-64 or HEK293 cells was ablated by addition of DNAse I (Figure 7C), we hypothesized that HpARI binds directly to DNA in the nucleus of necrotic epithelial cells.

*In vivo*, DNAse co-administration with *Alternaria* allergen abrogated HpARI suppression of IL-33 as measured by western blot, but not by ELISA, in the latter case presumably due to steric hindrance of ELISA antibodies on released HpARI-bound IL-33 (Figures 7D and 7E). We conclude that dual binding of DNA and IL-33 by HpARI results in retention of IL-33 within the necrotic cell nucleus, conferring a tethering function on HpARI in addition to its ability to block IL-33 in the fluid phase.

Binding of DNA by HpARI was confirmed using a gel shift assay, in which addition of HpARI retarded the migration of linear plasmid DNA through an agarose gel in a concentration-dependent manner (Figure 7F), and by immunoprecipitation of plasmid DNA by HpARI (Figure S5D). We hypothesised that HpARI could bind DNA through electrostatic interactions, as shown for other CCP module-containing proteins (Sjöberg et al., 2007, Trouw et al., 2005). When the isoelectric point (pI) of each of the three CCP domains of HpARI were calculated, CCP2 and CCP3 were found to be relatively acidic (pI 6.32 and 5.34 respectively), while CCP1 was strongly basic (pI 9.79). Indeed, an electrostatic surface representation of our 3-D model of CCP1 (Figure S5E), reveals clusters of solvent-exposed positively charged residues that could serve as a binding site for oppositely-charged (acidic) DNA. We produced truncated versions of HpARI, either encoding CCP1/2 or CCP2/3. As predicted, we found that only the CCP1/2 truncation caused a shift in DNA migration (Figure 7F), supporting a role for CCP1 in binding to DNA.

*In vivo*, only the CCP1/2 HpARI truncation could inhibit the release of IL-33 as measured by western blot, while CCP2/3 actually increased total quantities of IL-33 detected in the BAL (Figure 7G). Both constructs suppressed IL-33 detection by ELISA (Figure 7H), indicating they could both bind IL-33 and inhibit binding of ELISA antibodies. Therefore we propose that CCP2/3 does not inhibit IL-33 release but instead binds it in solution, prevent it from being degraded or taken up via its receptor. This data supports a model by which HpARI binds to IL-33 through its CCP2 domain, and to DNA through its CCP1 domain, tethering IL-33 within the necrotic cell nucleus.

## Discussion

IL-33 has emerged as a critical initiator of allergic responses in diseases such as asthma, sparking an array of type 2 reactions in innate lymphoid cells, eosinophils, macrophages, and T cells (Liew et al., 2016). Through screening of the secreted products of a helminth parasite we identified HpARI, a CCP module-containing protein that inhibits IL-33 release. Recombinant HpARI is non-cell permeable, and can only gain access to the nucleus of necrotic cells, where it binds directly to IL-33 and nuclear DNA, tethering IL-33 within necrotic cells and preventing binding to the IL-33R, thereby suppressing ILC2 responses and eosinophilia in the lung after *Alternaria* administration.

The primary mechanistic effect of HpARI is to bind IL-33: remarkably, this extends from murine to human IL-33. Although the affinity of HpARI for human IL-33 is lower than that of mouse IL-33, this binding is sufficient to prevent human IL-33 release, with a reduced IL-33 signal in human lung explant supernatants when cultured with HpARI, and reduced human IL-33 release in the lungs of human IL-33 transgenic mice. In the mouse, HpARI proved to be highly suppressive *in vivo*, recapitulating and exceeding the effects of total parasite secretions (HES), and able to inhibit IL-33 release even when administered 24 hr prior to allergen challenge.

Although it is clear that IL-33 is released at high levels during tissue injury and necrosis, it is presently unclear how IL-33 is secreted during homeostasis (Liew et al., 2016). We showed that HpARI was not able to penetrate intact cells thus, in the absence of cell membrane damage, HpARI would be unable to mediate the nuclear retention of IL-33. HpARI’s unique mechanism of action and specificity provide an interesting tool to investigate the role of IL-33 as an alarmin—preventing the release of IL-33 from necrotic cells while leaving other responses (necrosis, HMGB1 or IL-1α release) unaffected. Recently, IL-33 production and release by activated mast cells in response to extracellular ATP release was demonstrated in *H. polygyrus* infection (Shimokawa et al., 2017), and extracellular ATP has previously been shown to induce IL-33 release in response to *Alternaria* administration (Kouzaki et al., 2011). These findings might explain the lack of total ablation of IL-33 release with HpARI administration, as some cytokine might be actively secreted by live mast cells, against which the tethering function of HpARI would be inactive, without exposed DNA in a necrotic, lysed cell. In this context, the role of *H. polygyrus* secreted apyrases (Hewitson et al., 2011)—enzymes which degrade extracellular ATP—might have a further role.

Binding to nuclear DNA allows HpARI to hold active IL-33 within the necrotic cell, and ablates allergic sensitization. Although the affinity for DNA was not determined in this study, evidence from gel shift and co-immunoprecipitation assays, as well as ablation of necrotic nuclear localization and IL-33 tethering function on DNAse treatment, strongly supports binding of HpARI to DNA. Truncated HpARI lacking CCP1 has no activity in the gel shift assay and lacks IL-33 tethering functionality, and molecular modeling of CCP1 revealed 2 exposed basic patches as putative DNA binding sites. Of note, the mammalian CCP domain-containing proteins C4b-binding protein (C4BP) (Trouw et al., 2005) and complement factor H (Leffler et al., 2010), also bind DNA through basic CCP modules. The importance of IL-33 localization to the nucleus has been shown in transgenic mice lacking the nuclear localization domain of IL-33, which develop lethal eosinophil-dominant multi-organ inflammation (Bessa et al., 2014), and in human endothelial cells, where extracellular IL-33 leads to inflammatory responses, while nuclear IL-33 does not (Gautier et al., 2016).

Three predicted CCP modules span the length of mature HpARI. CCP module-containing proteins are present in different phyla including chordates and nematodes, with notable expansion and diversification in parasitic species such as *H. polygyrus* (Hewitson et al., 2013). The functions of CCP modules are diverse, underlining the versatility of this structural scaffold that has evolved to serve many purposes (Kirkitadze and Barlow, 2001, Soares and Barlow, 2005, Soares et al., 2005). Of note, no non-host CCP module-containing protein has previously been shown to have immunomodulatory function outside of the complement system, and hence the co-option of this module by a parasite to block a mammalian immunological pathway is remarkable.

The suppression of the IL-33 pathway by *H. polygyrus* at the level of the IL-33 cytokine (mediated by HpARI) and the IL-33 receptor (mediated by secreted exosomes [Buck et al., 2014]) indicates that this pathway might be critical to persistence of the parasite. Indeed administration of exogenous IL-33 induces expulsion of *H. polygyrus* (Yang et al., 2013), while IL-33R-deficient mice are slow to expel this parasite even when immunized with a vaccine that induces sterile immunity in wild-type mice (Coakley et al., 2017). Similarly, in many helminth infections IL-33 administration can drive immunity, while deficiency of IL-33 or the IL-33 receptor leads to increased parasite load (Maizels and McSorley, 2016). Hence, the ability of *H. polygyrus* to pre-empt the IL-33 alarmin system is likely to be a pivotal evolutionary adaptation to allow establishment in the mammalian host.

HpARI administration suppressed the eosinophilic response to *N. brasiliensis* infection, leading to reduced ejection of adult parasites from the intestinal lumen, similarly to the phenotype seen in IL-33-deficient animals (Hung et al., 2013). Thus HpARI is capable of suppressing early innate anti-parasite immunity, a role we hypothesize it to play in the early stages of *H. polygyrus* infection where IL-33 is critical for resistance (Coakley et al., 2017).

During an *H. polygyrus* infection, larvae penetrate the gut wall, undergo two molts in the subserosal membrane, and emerge back into the lumen of the gut as adults (Maizels et al., 2012). As the parasite penetrates the intestinal wall, it damages epithelial cells which could result in the release of pre-formed IL-33 and induction of a parasite-toxic type 2 immune response. HpARI is secreted by the parasite larvae and adult (Hewitson et al., 2013) and so is well positioned to ablate this IL-33 response.

Recently, IL-33 was implicated in activation of intestinal Foxp3^+^ regulatory T (Treg) cells (Schiering et al., 2014) raising the possibility that HpARI could interfere with Treg cell-mediated suppression. However, in mouse models of asthma, IL-33 signaling to IL-33R^+^Foxp3^+^ Treg cells results in their expression of Th2 cytokines, and abrogation of suppressive ability (Chen et al., 2017). Thus, in asthmatic responses at least, IL-33 appears to have an inflammatory, rather than suppressive effect.

In conclusion, we have identified a CCP module-containing protein with the unique ability to selectively bind to IL-33 and DNA within necrotic epithelial cells. This activity potently suppresses the release and the biological activity of IL-33, resulting in suppression of type 2 responses to allergen challenge. IL-33 is a critical mediator in allergic disease and an important clinical target. HpARI could be a potent agent for prevention of IL-33-mediated pathology, as well as a new tool for manipulation of IL-33 release, leading to better understanding of the IL-33 pathway.

## STAR★Methods

### Key Resources Table

**Table:** 

REAGENT or RESOURCE	SOURCE	IDENTIFIER
**Antibodies**		

Anti-mouse CD3 (clone 145-2C11)	Biolegend	100306
Anti-mouse CD4 (clone RM4.5)	Biolegend	100566
Anti-mouse CD5 (clone 53-7.3)	Biolegend	100606
Anti-mouse CD11b (clone M1/70)	Biolegend	101224
Anti-mouse CD11c (clone N418)	Biolegend	117312
Anti-mouse CD19 (clone 6D5)	Biolegend	11506
Anti-mouse CD25 (clone PC61)	Biolegend	102038
Anti-mouse CD45 (clone 30-F11)	Biolegend	103128
Anti-mouse CD49b (clone DX5)	eBioscience	11-5971-85
Anti-mouse CD127 (clone A7R34)	Biolegend	135013
Anti-mouse ICOS (clone 15F9	eBioscience	46-9940-82
Anti-mouse GR1 (clone RB6-8C5)	Biolegend	108406
Anti-mouse IL-5 (clone TRFK5	Biolegend	504304
Anti-mouse IL-13 (clone eBio13A)	eBioscience	25-7133-82
Anti-mouse Ly6G (clone 1A8)	Biolegend	127616
Anti-mouse SiglecF (clone ES22-10D8)	Miltenyi	130-102-274
Anti-mouse ST2 (clone RMST2-2)	eBioscience	17-9335-82
Anti-mouse TER119 (clone TER-119)	Biolegend	116220
Anti-HMGB-1 rabbit polyclonal	Abcam	Ab18256
Anti-c-myc (clone Myc.A7)	Thermo Fisher Scientific	MA1-21316
Anti-human IL-33 goat polyclonal	R&D Systems	AF3625
Anti-mouse IL-33 goat polyclonal	R&D Systems	AF3626
Anti-mouse IL-1α	R&D Systems	AF-400-NA
IgG1 isotype control antibody (clone MOPC-21)	Produced in-house	N/A

**Bacterial and Virus Strains**		

*Heligmosomoides polygyrus*	(Johnston et al, 2015)	N/A
*Nippostrongylus brasiliensis*	(Lawrence et al, 1996)	N/A

**Biological Samples**		

Human lung tissue	Lothian NRS Bioresource	15/ES/0094

**Chemicals, Peptides, and Recombinant Proteins**		

Recombinant mouse IL-1α	Biolegend	575002
Recombinant mouse IL-33	Biolegend	580506
Recombinant human IL-33	Biolegend	581806
ST2-Fc	Biolegend	557904
Dynabeads Protein G	Thermo Fisher Scientific	10004D
Proteinase K	Sigma	557904
DNAse (protease-free)	Sigma	4536282001
Liberase TL	Sigma	05401020001
Methylcholine chloride	Sigma	A2251
Hoescht 33342	Thermo Fisher Scientific	H3570

**Critical Commercial Assays**		

Mouse IL-33 Duoset ELISA	R&D systems	DY3626
Human IL-33 Duoset ELISA	R&D systems	DY3625B
Annexin V Apoptosis Detection Kit	eBioscience	88-8005-72
Limulus Amoebocyte Lysate assay	Lonza	QCL-1000

**Experimental Models: Cell Lines**		

HEK293T	ATCC	CRL-3216
CMT-64	ECACC	10032301

**Experimental Models: Organisms/Strains**		

Mouse: IL-13-eGFP (C57BL/6J)	(Neill et al., 2010)	N/A
Mouse: hIL-33^+/+^ / mIL-33^–/–^ (humanized IL-33) (BALB/c)	(Cohen et al., 2015)	N/A

**Recombinant DNA**		

pSecTAG2A plasmid	Thermo Fisher Scientific	V90020
**Software and Algorithms**		
ClustalX	(Thompson et al., 1997)	www.clustal.org
Mascot v2.4	Matrix Science	www.matrixscience.com
SMART	(Letunic et al., 2015)	smart.embl-heidelberg.de/
HHpred	(Söding, 2005)	toolkit.tuebingen.mpg.de/#/tools/hhpred
Modeller v9.12	(Sali and Blundell, 1993)	salilab.org/modeller/
APBS	(Baker et al, 2001)	www.poissonboltzmann.org/
ESPript v3	(Robert and Gouet, 2014)	espript.ibcp.fr/
PyMOL	Schrödinger, LLC	www.pymol.org
PROSITE	(de Castro et al., 2006)	prosite.expasy.org/
Protein Data Bank	(Berman et al., 2000)	www.rcsb.org/pdb
Wormbase ParaSite	(Howe et al., 2016)	parasite.wormbase.org/
FlowJo v9.1	Flowjo, LLC	www.flowjo.com/
Prism v7	Graphpad Software	www.graphpad.com/scientific-software/prism/
BIAcore T200 software v2.01	GE Healthcare	N/A

**Other**		

Superdex 200 10/300 GL	GE Healthcare	17517501
MonoQ 5/50 GL	GE Healthcare	17-5166-01
Series S Sensor Chip NTA	GE Healthcare	BR-1005-32

### Contact for Reagent and Resource Sharing

Further information and request for resources and reagents should be directed to and will be fulfilled by the Lead Contact, Henry McSorley (henry.mcsorley@ed.ac.uk).

### Experimental Model and Subject Details

#### Mice

BALB/cOlaHsd, C57BL/6JOlaHsd, IL-13-eGFP (C57BL/6 background) (Neill et al., 2010) and ST2-deficient (BALB/c background, kindly provided by Dr Andrew McKenzie, MRC Laboratory of Molecular Biology, Cambridge) mice, male or female (single sex within an experiment), 6-10 weeks old, were bred in-house at the University of Edinburgh. hIL-33^+/+^, mIL-33^–/–^ (humanised IL-33) transgenic mice (BALB/c background) (Cohen et al., 2015) were bred in-house at the Babraham Institute, Cambridge. All mice were accommodated, and procedures performed under UK Home Office licenses with institutional oversight performed by qualified veterinarians.

#### Human Tissue Samples

Non-cancerous adjacent tissue from lung cancer patients was collected by Lothian NRS Bioresource, and cultured as previously described (Cohen et al., 2015). The study was approved by Lothian NRS Bioresource (15/ES/0094) and tissue was donated with the informed consent of patients.

### Method Details

#### Parasite lifecycles, Infection, and HES Preparation

The life cycle of *H. polygyrus bakeri* was maintained, and HES products prepared, as previously described (Johnston et al., 2015). The life cycle of *N. brasiliensis* was maintained in Sprague-Dawley rats as previously described (Lawrence et al., 1996), and infective L3 larvae were prepared from 1-3 week rat fecal cultures. BALB/c mice were subcutaneously infected with 500 L3 *N. brasiliensis* larvae. At day 3 post-infection, larvae were counted in the bronchoalveolar lavage and in lung tissue, by dicing lungs and placing them in a cheese-cloth bag in a 50 ml tube containing PBS at 37°C for at least 3 h. Day 3 lung counts reflect a sum of the BAL and lung larval counts for each animal. At day 6 intestinal worms were recovered from intestinal tissue using an adapted Baermann apparatus.

#### Reagents

*Alternaria alternata* extract (Greer XPM1D3A25) was resuspended in PBS, filter sterilized and concentration assessed by BCA assay (Pierce). CMT-64 cells (ECACC 10032301) and HEK293T cells (ATCC CRL-3216) were maintained by serial passage in DMEM medium containing 10% fetal bovine serum, 2 mM L-glutamine and 1 μg ml^-1^ penicillin/streptomycin. Human and murine IL-33 and murine IL-1α were purchased from BioLegend.

#### In Vitro IL-33 Release Assay

HES, candidate proteins or HpARI were cultured with total murine lung cells prepared by Liberase/DNAse digestion of naïve mouse lungs or CMT-64 cells for 1 h at 37°C, 5% CO_2_, with *Alternaria* allergen (200 μg ml^-1^), or were frozen on dry ice, and thawed at 37°C.

#### Preparation of Murine Lung Single Cell Suspension

Single-cell suspensions of naïve murine lung tissue were prepared by digesting in 2 U ml^-1^ liberase TL (Roche, Burgess Hill, UK) and 80 U ml^-1^ DNase (Life Technologies, Paisley, UK) at 37°C with agitation for 35 min. Digested tissue was macerated through a 70 μm cell strainer (BD Biosciences), treated with red blood cells lysis buffer (Sigma), and live cells counted on a haemocytometer, excluding dead cells by trypan blue staining.

#### Cytokine Measurement

R&D Systems Duoset kits were used to measure human and murine IL-33 by ELISA, while western blotting was carried out using polyclonal goat anti-mouse IL-33, goat anti-human IL-33 or goat anti-mouse IL-1α (R&D Systems) with a rabbit anti-goat IgG HRP secondary antibody (Thermo Fisher), and detected using WesternSure Premium reagent (Licor).

#### Fractionation and Mass Spectrometry

HES was separated into 1 ml fractions by size exclusion chromatography using a Superdex 200 10/300 GL column, or by anion exchange chromatography using a MonoQ 5/50 GL column (GE Healthcare) in a 40 column volume gradient from 20 mM TrisHCl pH 8 (start buffer) to a maximum of 30% 20 mM TrisHCl + 1 M NaCl pH 8 (elution buffer). All fractions were trypsinized and analyzed by LC MS/MS on an on-line system consisting of a capillary-pump Agilent 1200 HPLC system (Agilent, UK) coupled to an Orbitrap XL mass spectrometer (Thermo Scientific) as previously described (Hewitson et al., 2011, Hewitson et al., 2013). LC MS/MS data was analyzed using Mascot (v2.4, Matrix Science) and searched against an improved in-house BLASTx annotated database obtained by 454 sequencing of *H. polygyrus* adults, with additional full length *H. polygyrus* sequences from NCBI, WormBase ParaSite (Howe et al., 2016) and our own Sanger sequencing (Harcus Y. et al, manuscript in preparation). Peptides identified were ranked by Mascot protein score, with a minimum cutoff score of 20, with a significance threshold of p<0.05. Protein abundance was estimated by emPAI (exponentially modified protein abundance index).

#### Protein Expression and Purification

Candidate genes were selected by comparison of emPAI and IL-33-suppression profiles in all fractions (Figures S1 and S2). Candidate genes A-D (Figure 2A, respectively Hp_I10793_IG03481_L623, Hp_I15874_IG07818_L1106, Hp_I46029_IG37973_L313 and Hp_I08176_IG02172_L1157 transcripts) were codon optimised for *Homo sapiens* and gene synthesised (GeneArt, Thermo Fisher) with 5’ AscI and 3’ NotI restriction enzyme sites. CCP1/2 (amino acids 17-165) and CCP2/3 (amino acids 80-251) constructs were created using PCR of codon-optimised HpARI, and primers which added a NotI site 3’ of the CCP2 module (5’GCGGCCGCCTTGGGGCACACGCCCAG3’, primes reverse of LGVCPK amino acid sequence, for CCP1/2 construct), or an AscI site 5’ of the CCP2 module (5’ 5’GGCGCGCCGGCTGCAAGGGCATCCTG3’, primes GCKGIL amino acid sequence, for CCP2/3 construct), combined with vector-specific T7 (5’ of insert) and BGH (3’ of insert) primers. The HpARI_mCherry fusion protein was created by cloning in a codon-optimised gene-synthesised mCherry sequence (ANO45948.1) at the C-terminus of the HpARI protein, using an mCherry 5’ NotI site and a 3’ ApaI site. These constructs were sub-cloned into the pSecTAG2A expression vector (Thermo Fisher), using AscI, NotI-HF and Apa-1 restriction enzyme digestion (New England Biolabs), followed by T4 DNA ligation (Thermo Fisher).

JM109 cells were transformed with ligated constructs and plasmids were midiprepped using the PureLink HiPure midiprep kit (Thermo Fisher) according to manufacturer’s instructions, and Sanger sequenced. Plasmid constructs were transfected into HEK293T cells using the calcium phosphate technique (Jordan et al., 1996), using 15 μg plasmid DNA per 100 mm tissue culture dish of HEK293T cells at 20% confluency. Stable cell lines were maintained using Zeocin (Thermo Fisher) selection in DMEM medium containing 10% fetal bovine serum, 2 mM L-glutamine and 1 μg ml^-1^ Penicillin/Streptomycin.

Resulting expressed proteins secreted to the medium contained C-terminal myc and 6-His tags. For large scale expression of constructs, transfected cells were transferred to 293 SFM II media (Thermo Fisher) and protein purified from supernatant by nickel affinity chromatography using HiTrap chelating HP columns (GE Healthcare), eluting bound proteins using an imidazole gradient. Fractions containing pure expressed protein were pooled, dialysed into PBS, sterile filtered and concentration assessed by absorbance at 280 nm, corrected by calculated extinction coefficient.

Purified HpARI had an endotoxin content of below 0.5 U LPS per μg protein, as measured by the Limulus Amoebocyte Lysate assay (Lonza).

#### Bioinformatics Characterization and Modeling

Domain identification and assignment were undertaken using a combination of SMART (Letunic et al., 2015), an HHPred search against the pdb70 database (accessed March 2016) (Berman et al., 2000, Söding, 2005), and refined manually based upon positioning of the four Cysteine residues that typify CCP module sequences (Soares et al., 2005). PROSITE (de Castro et al., 2006) was used for short motif searches. ESPript v3 (Robert and Gouet, 2014) was used for alignment figure preparation.

The three predicted HpARI CCP module sequences were modeled based upon their top ranked CCP module template structure ‘hits’ as suggested by HHPred. HpARI-CCP1 was modeled based upon CR2-CCP2 (PDB ID: 1LY2) (Prota et al., 2002) (after a manual switch of Leu^69^ with Trp^69^ to help identify this CCP module using HHPred; note Leu/Trp substitutions exist in other experimentally-determined CCP module structures such as complement Factor H CCP10 and CCP20 (Makou et al., 2012, Morgan et al., 2012); HpARI-CCP2 on CSMD1-CCP3 (PDB ID: 2EHF) (RIKEN Structural Genomics/Proteomics Initiative); HpARI-CCP3 on GABABR1α-CCP2 (PDB ID: 1SRZ) (Blein et al., 2004). The target-template alignment in each case was based upon the initial HHPred alignment, then extended to include the first Cysteine residue in each domain, realigned using ClustalX (Thompson et al., 1997), and finally subjected to manual editing to optimally position known consensus residues, secondary structure elements and gaps (Soares et al., 2005). Note, an alternative alignment for the atypical insertion in CCP3 is possible where it can be accommodated after the hypervariable loop (not shown). A total of 100 models for each CCP module were built using Modeller v9.12 (Sali and Blundell, 1993), and the model with the lowest DOPE (Shen and Sali, 2006) energy score selected as the representative model in each case and evaluated for valid stereochemistry (Lovell et al., 2003). Electrostatic surface potential was calculated using APBS (Baker et al, 2001). PyMOL (http://www.pymol.org/; Schrödinger, LLC.) was used for visualization, and figure preparation.

#### Alternaria Models

*Alternaria* models, lung cell preparation, flow cytometry and lung histology were carried out as previously described (McSorley et al., 2014). *Alternaria* allergen (25 μg) was administered intranasally with 20 μg OVA protein (Sigma) and HpARI (10 μg). In some experiments, the OVA-specific response was recalled by daily intranasal administration of 20 μg OVA protein on days 14, 15 and 16. Mice were culled 15 min, 1 h, 24 h or 17 days after the initial administration, as indicated. Bronchoalveloar lavage was collected (4 lavages with 0.5 ml ice-cold PBS), followed by lung dissection for tissue digestion and single cell preparation (see below), or lungs were inflated with 10% neutral buffered formalin for histology. Formalin-fixed lungs were transferred into 70% ethanol 24 h after collection, paraffin, embedded and sectioned (5 μm), prior to staining with haemotoxylin and eosin (H&E) or Periodic Acid Schiff (PAS). H&E and PAS-stained sections were scored blindly according to the following criteria: H&E stain at 200X magnification on an increasing severity score of 1–4 in both the peri-vascular and peri-bronchiolar compartments (1 = <5, 2 = 5-20, 3 = 20-100, 4 = >100 cells), giving an average overall score of 5-10 fields of view per section. PAS stained sections were scored at 100X magnification, on percentage of mucous-positive epithelial cells (1 = <1%, 2 = 1-20%, 3 = 20-50%, 4 = 50-100%), of 5-10 fields of view per section.

#### Measurement of Airway Hyperresponsiveness

A Flexivent system (Scireq, Montreal, Canada) was used to measure dynamic resistance and compliance. Mice were anaesthetised with intraperitoneal ketamine 200 mg/kg and pentobarbitone (50 mg/kg), tracheotomised and mechanically ventillated. Lung resistance and compliance were measured in response to nebulised methacholine (Sigma).

#### Immunoprecipitation

Protein G dynabeads (Thermo Fisher) were coated with 5 μg anti-c-Myc (clone Myc.A7, Thermo Fisher), MOPC (IgG1 isotype control antibody) or ST2-Fc fusion protein (Biolegend), and washed on a DynaMag-2 magnet with PBS containing 0.02% Tween 20. These were then used to immunoprecipitate HpARI-IL-33 complexes, following manufacturer’s instructions.

Where human or mouse lung homogenates were used, these were prepared by homogenizing (Tissuelyser II, QIAGEN) one lung lobe (mouse) in 1 ml PBS, or 400 mg human lung tissue in 1 ml PBS. Lung homogenates (100 ul) or 100 ng human or murine recombinant IL-33 (Biolegend) were then mixed with 1 μg HpARI in PBS containing 100 ug/ml OVA protein, and incubated for 30 min at 37°C. Complexes were then added to coated dynabeads, incubated for 10 min at room temperature, and unbound material collected. Bound material on beads was washed 3 times in PBS+0.02% PBS on a DynaMag-2 magnet, before transferring to a fresh tube and eluting bound complexes using 50 mM glycine pH 2.8 (non-denaturing), before neutralising in 1M Tris buffer, pH 8. Eluted proteins and unbound supernatants were ran on 4-12% SDS-PAGE gels (Thermo Fisher) under non-reducing conditions, and transferred to nitrocellulose membranes for western blotting.

#### Surface Plasmon Resonance (SPR)

SPR measurements were performed using a BIAcore T200 instrument (GE Healthcare). Ni^2+^-nitrilotriacetic acid (NTA) sensor chips were purchased from GE Healthcare. HpARI was immobilised on an NTA sensor surface to 400 RU, which gave essential zero baseline drift over the time course of the experiments performed: the apparent k- for the His-tagged HpARI Ni^2+^-NTA surfaces was significantly slower than the complex being studied ∼5×10^-5^ s^-1^ for HpARI-Ni-NTA *vs* ∼14-400×10^-5^ s^-1^ for HpARI-IL-33, therefore short cycle (400-600 s total run times) single kinetic analysis could be reliably performed. Following Ni^2+^ priming (30 sec injection of 500 μM NiCl_2_ at 5 μl·min^-1^) 50 nM HpARI, in 10 mM NaH_2_PO_4_, pH 7.5; 150 mM NaCl; 50 μM EDTA; 0.05% surfactant P20, was captured *via* the 6-His tag by injection for 15 seconds, at 30 μl·min^-1^. Surface regeneration between cycles and/or experiments was performed by dissociating any immobilised His-tagged protein or complex by a 90 s injection of 350 mM EDTA, in 10 mM NaH_2_PO_4_, pH 7.5; 150 mM NaCl; 0.05% surfactant P20 followed by a 30 s injection of 50 mM NaOH at the same flow rate.

SPR kinetic titration binding experiments were performed at 25°C. Three-fold dilution series of mIL-33 (6.2 nM to 167 nM) or hIL-33 (0.062 μM to 1.67 μM), were injected over the sensor surface, in 10 mM NaH_2_PO_4_, pH 7.5; 150 mM NaCl; 50 μM EDTA; 0.05% surfactant P20, at 30 μl.min^-1^ for 30 s followed by a 60 s dissociation phase. The same concentration series of mIL-33/hIL-33 were ran over Ni^2+^-charged NTA surfaces, and showed no evidence of non-specific interaction of mIL-33/hIL-33 interacting with these surfaces. All experiments were performed on Ni^2+^-charged surfaces following non-specific binding assessment and were double referenced using similar blank surface responses for run-noise corrections. The on- (*k*_+_) and off-rate (*k*_–_) constants and the equilibrium dissociation constant (KD) were calculated by global fitting all three surfaces simultaneously to a 1:1 interaction model, with mass transport considerations, to the double reference corrected sensorgrams, using analysis software (v.2.01, GE Healthcare) provided with the BIAcore T200 instrument.

Both interactions were extremely well fit by a simple 1:1 interaction model (Chi^2^ values of 0.457 and 0.395, mIL-33 and hIL-33 respectively), with RUmax values close to the theoretical maximum expected for a 1:1 stoichiometric interaction with high specific activity (∼ 180 RU; 173 RU and 169.3 RU, mIL-33 and hIL-33 respectively) and showed no evidence of mass transport issues.

#### Human Lung Explant Culture

Approximately 5 g of lung tissue was washed 3 times in PBS and ∼0.5 mm^2^ tissue explants prepared using sterilized scissors. Explants were incubated in 400 μl PBS+0.1% BSA +/- 10 μg/ml HpARI in wells of a 48-well tissue culture plate (Costar) for 1 h, at 37°C, 5% CO_2_. Each condition was performed with 8 replicates for IL-33 measurement by ELISA, and pairs of supernatants were pooled (to make 4 replicates) for IL-33 western blot. After culture, tissue pieces were weighed, and IL-33 levels calculated relative to tissue weight.

#### Gel Shift Assay

Linearized Not-HF-cut pSecTAG2A plasmid (10 ng) was mixed with HpARI, CCP1/2 and CCP2/3 proteins, in 10 mM TrisCl, 1 mM EDTA, and incubated for 30 min at 37°C. Complexes were ran on a 0.7% agarose gel and imaged with Gelred (Biotium).

### Quantification and Statistical Analysis

All data was analyzed using Prism (Graphpad Software Inc.). Where two groups were compared, Student’s t-test was used, where there were 3 or more groups, one-way ANOVA with a Bonferroni’s post test was used, and for comparing groups at multiple timepoints two-way ANOVA with a Sidak’s post test was used. ^∗∗∗∗^ = p<0.0001, ^∗∗∗^ = p<0.001, ^∗∗^ = p<0.01, ^∗^ = p<0.05, N.S. = Not Significant (p>0.05).

### Data and Software Availability

The accession number for the HpARI transcript sequence as reported in this paper is Wormbase Parasite: HPBE_0000813301.

## Author Contributions

M.O., F.V., E.S.C., I.C.S., W.F.G., M.T., A.M.K., D.J.S., H.H., A.G., C.E., and H.J.M. carried out the experiments. D.C.S., A.M.K., T.B., M.W., and A.C.I. carried out bioinformatic and structural analyses. A.M.K., K.J.F., J.P.H., and H.J.M. designed and carried out fractionation experiments. W.A.W. provided human tissue. E.S.C., I.C.S., S.V., A.L.A., J.S., R.M.M., and H.J.M. designed the experiments. M.O., D.C.S., E.S.C., I.C.S., R.M.M., and H.J.M. wrote the manuscript.

## Supplemental Information

Document S1. Figures S1–S5 and Table S1

Document S2. Article plus Supplemental Information
