# Supplementary material for: HpARI Protein Secreted by a Helminth Parasite Suppresses Interleukin-33
Source: Immunity. 2017 Oct 17;47(4):739–751.e5. doi: 10.1016/j.immuni.2017.09.015 (PMC5655542; doi:10.1016/j.immuni.2017.09.015)
Supplement: Document S1. Figures S1–S5 and Table S1 [file mmc1.pdf]

Immunity, Volume *47*

Supplemental Information

HpARI Protein Secreted by a Helminth Parasite Suppresses Interleukin-33

Megan Osbourn, Dinesh C. Soares, Francesco Vacca, E. Suzanne Cohen, Ian C. Scott, William F. Gregory, Danielle J. Smyth, Matilda Toivakka, Andrea M. Kemter, Thierry le Bihan, Martin Wear, Dennis Hoving, Kara J. Filbey, James P. Hewitson, Holly Henderson, Andrea Gonzàlez-Cìscar, Claire Errington, Sonja Vermeren, Anne L. Astier, William A. Wallace, Jürgen Schwarze, Alasdair C. Ivens, Rick M. Maizels, and Henry J. McSorley

	Blast hit	Size fraction 11 emPAI	Mascot protein score	Rank (of 194) [a]	Charge fraction 25 emPAI	Mascot protein score	Rank (of 262) [a]
Hp_I10793_IG03481_L623	No BLASTX similarities 13330478:13331100 forward	1.35	99	57	0.53	104	77
Hp_I15874_IG07818_L1106	BLASTX similarity to 66.67% ID to CBN-NUC-1 protein [Caenorhabditis brenneri] (376 aa, Accession: gi|341901954|gb|EGT57889.1|)	1.11	229	19	5.23	786	2
Hp_I46029_IG37973_L313	BLASTX similarity to 55.93% ID to unnamed protein product [Homo sapiens] (304 aa, Accession: gi|189069304|dbj|BAG36336.1|)	2.01	67	90	0.73	50	163
Hp_I08176_IG02172_L1157	BLASTX similarity to 72.38% ID to PHA domain [Heligmosomoides polygyrus bakeri] (252 aa, Accession: gi|345499002|emb|CCC54333.1|)	0.13	61	100	1.05	353	18
GSXTT4C07IB13H_length=463	BLASTX similarity to 86.32% ID to venom allergen/ ancylostoma secreted protein-like 1 isoform 4 [Heligmosomoides polygyrus bakeri] (459 aa, Accession: gi|348659354|gb|AEP82914.1|)	0.53	59	105	2.58	246	1
Hp_I00796_IG00050_L1763	BLASTX similarity to 60.51% ID to metalloprotease 1 precursor [Ancylostoma ceylanicum] (547 aa, Accession: gi|23268453|gb|AAN11401.1|)	0.37	163	32	0.78	518	3
Hp_I03365_IG00388_L986	BLASTX similarity to 83.05% ID to putative ES protein F7 [Ostertagia ostertagi] (181 aa, Accession: gi|18104159|emb|CAD20464.1|)	0.2	73	80	3.37	408	14
Hp_I05355_IG00918_L1570	BLASTX similarity to 61.84% ID to astacin-like metalloprotease [Haemonchus contortus] (502 aa, Accession: gi|82653303|emb|CAJ43810.1|)	0.51	205	20	0.34	137	20
Hp_I07496_IG01832_L2183	BLASTX similarity to 49.47% ID to metalloprotease I [Ostertagia ostertagi] (573 aa, Accession: gi|25005280|emb|CAD28559.2|)	0.23	184	24	0.52	300	26
Hp_I09193_IG02681_L996	BLASTX similarity to 27.72% ID to Chain A, Caclcium-Bound Ac-Asp-7 (206 aa, Accession: gi|383875397|pdb|3S6U|A)	0.2	78	72	1.28	312	24
Hp_I09338_IG02753_L893	BLASTX similarity to 69.57% ID to Chain A, Glutathione Transferase-2, Apo Form, From The Nematode Heligmosomoides Polygyrus (206 aa, Accession: gi|51247756|pdb|1TW9|A)	0.36	81	60	0.23	90	59
Hp_I12757_IG04701_L2227	BLASTX similarity to 69.27% ID to hypothetical protein CAEBREN_19315 [Caenorhabditis brenneri] (713 aa, Accession: gi|341886485|gb|EGT42420.1|)	0.9	321	9	0.29	119	67
Hp_I12915_IG04859_L2071	BLASTX similarity to 74.56% ID to hypothetical protein CAEBREN_01953 [Caenorhabditis brenneri] (594 aa, Accession: gi|341889762|gb|EGT45697.1|)	0.25	146	39	0.43	190	39
Hp_I13075_IG05019_L1949	BLASTX similarity to 25.30% ID to GD14343 [Drosophila simulans] (699 aa, Accession: gi|195589672|ref|XP_002084573.1|)	0.71	317	10	1.15	478	8
Hp_I13426_IG05370_L1731	BLASTX similarity to 61.51% ID to hexokinase [Haemonchus contortus] (485 aa, Accession: gi|4583627|emb|CAB40412.1|)	1.51	564	4	0.81	409	13
Hp_I13832_IG05776_L1555	BLASTX similarity to 59.25% ID to metalloprotease III [Ostertagia ostertagi] (507 aa, Accession: gi|21425408|emb|CAD19995.2|)	0.6	152	38	0.9	340	20
Hp_I14648_IG06592_L1319	No BLASTX similarities 18271778:18273096 forward	1.67	280	15	0.75	180	41
Hp_I14766_IG06710_L1294	BLASTX similarity to 42.09% ID to Hyaluronidase-1, partial [Ascaris suum] (439 aa, Accession: gi|324516157|gb|ADY46439.1|)	0.15	57	109	0.42	96	86
Hp_I15012_IG06956_L1244	BLASTX similarity to 71.51% ID to hypothetical protein CRE_02222 [Caenorhabditis remanei] (368 aa, Accession: gi|308509410|ref|XP_003116888.1|)	0.56	167	21	0.25	138	57
Hp_I15720_IG07664_L1128	BLASTX similarity to 83.79% ID to hypothetical protein CRE_30062 [Caenorhabditis remanei] (425 aa, Accession: gi|308473183|ref|XP_003098817.1|)	0.5	180	26	0.28	88	99
Hp_I15761_IG07705_L1121	BLASTX similarity to 78.44% ID to hypothetical protein CAEBREN_23086 [Caenorhabditis brenneri] (322 aa, Accession: gi|341889746|gb|EGT45681.1|)	0.18	41	144	1.47	368	16
Hp_I16083_IG08027_L1071	BLASTX similarity to 28.20% ID to Complement factor H [Ascaris suum] (1358 aa, Accession: gi|324499597|gb|ADY39830.1|)	0.41	72	85	0.19	42	186
Hp_I28418_IG20362_L498	BLASTX similarity to 45.24% ID to hypothetical protein CRE_02231 [Caenorhabditis remanei] (147 aa, Accession: gi|308510374|ref|XP_003117370.1|)	0.43	42	141	0.43	67	122
Hpb-APY-2	No BLASTX analysis performed; (File Apyrase 124-3 (iso 07051) 081110) 1062 nt	5.9	654	2	0.3	58	137
Hpb-GST-2	No BLASTX analysis performed; partial (no N-terminus) AF128959	1.07	96	60	0.79	138	59

a) Rank of 194 proteins in size fraction 11 or 262 candidates in charge fraction 25 determined by Mascot score, with a minimum score of 20

Supplementary Table 1 (related to Figure 1):
Candidate genes with emPAI peaking in size fractions 10-12 and charge fraction 23-27. Data shown for peak IL-33 suppressive fraction (size = 11, charge = 25).

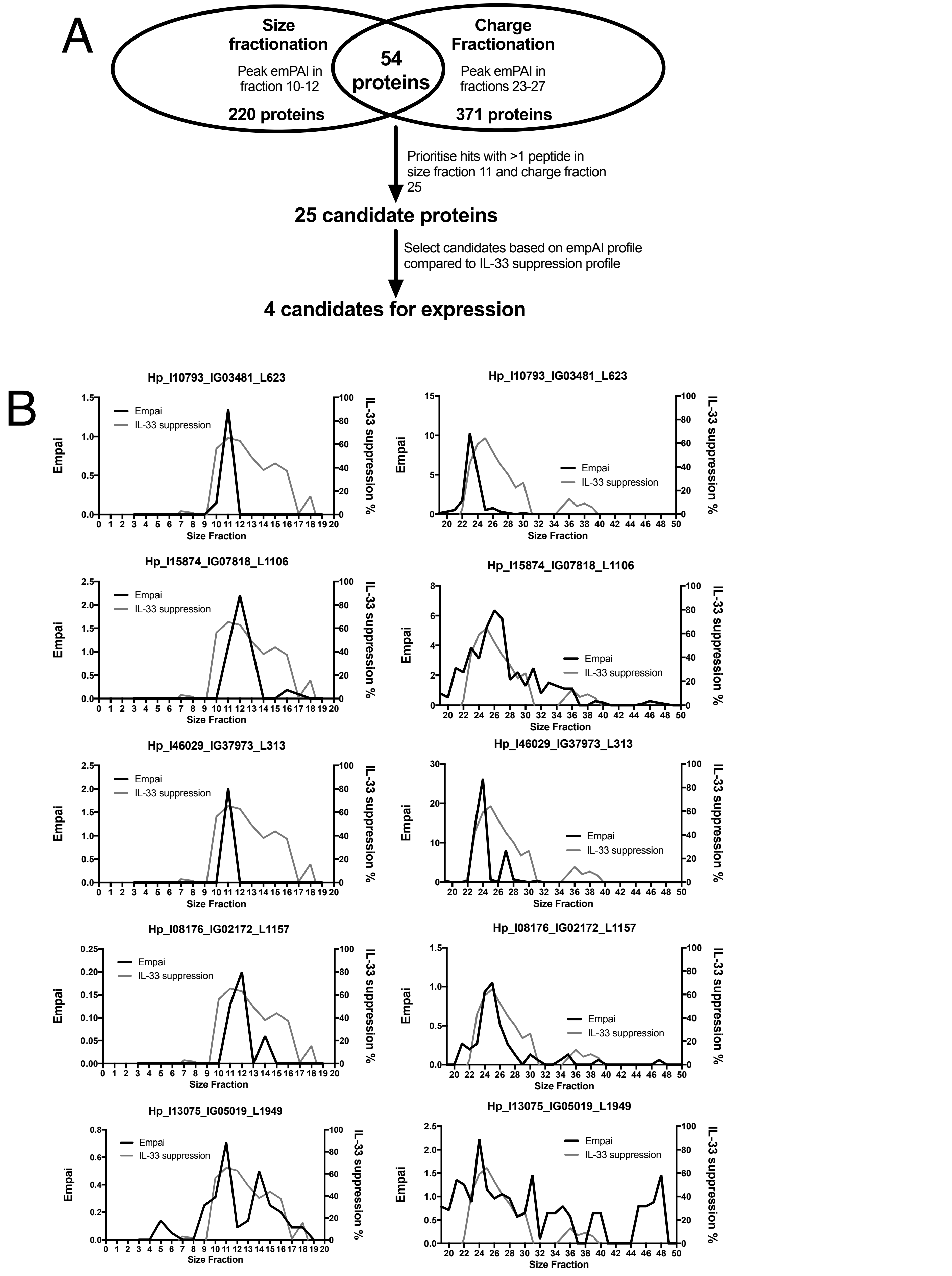

Supplementary Figure 1 (Related to Figure 1):

A. Candidate protein selection strategy
B. Candidate protein Empai profile and IL-33 suppression profile in HES size (left panels) and charge (right panels) fractions. The 4 candidate proteins tested in Fig 2A are shown followed by a candidate not selected for further testing (Hp_I13075_IG05019_L1949), for comparison.

A

```
0    atgtatcgcctttttctcgctttaggcttcctcaccttcatcaacgctgcaggtcagcgc   60
0    M  Y  R  L  F  L  A  L  G  F  L  T  F  I  N  A  A  G  Q  R     20

61   tgcagattcaccgatgtagaaagagacaaaggctacactggaatgctccttaaaggaagg   120
21   C  R  F  T  D  V  E  R  D  K  G  Y  T  G  M  L  L  K  G  R     40

121  ttaagaaagacagctggaaacgggaggacggtagaattgatatgtggaagaggaaatcac   180
41   L  R  K  T  A  G  N  G  R  T  V  E  L  I  C  G  R  G  N  H     60

181  aactacacctgcgagtccggcgttttgaaggaaagttctccacggcaagcccgatgtggt   240
61   N  Y  T  C  E  S  G  V  L  K  E  S  S  P  R  Q  A  R  C  G     80

241  tgcaagggaattctcgaaatgctcttcgatatgcctaaggaagaacggccctcgcccatg   300
81   C  K  G  I  L  E  M  L  F  D  M  P  K  E  E  R  P  S  P  M     100

301  tacgactcagtcacctatgaccccacgcccaacaccccaacaactgtaggaaaggacggg   360
101  Y  D  S  V  T  Y  D  P  T  P  N  T  P  T  T  V  G  K  D  G     120

361  atatggaacggagtagattaccgcaatggctctacagtgaagccttattgcgatactggt   420
121  I  W  N  G  V  D  Y  R  N  G  S  T  V  K  P  Y  C  D  T  G     140

421  ccagtcatcaacggatcatccaaagctgtatgtgtgagtggcaaatgggttcctacgctt   480
141  P  V  I  N  G  S  S  K  A  V  C  V  S  G  K  W  V  P  T  L     160

481  ggcgtctgcccaaaaatgtgttcgatcggcagcttgaaggaaaatgggaaattcgtagac   540
161  G  V  C  P  K  M  C  S  I  G  S  L  K  E  N  G  K  F  V  D     180

541  gtcacagcaacaactaaaggagacgagctcaacccccccgccaagggaacagacattgatt   600
181  V  T  A  T  T  K  G  D  E  L  N  P  P  P  R  E  Q  T  L  I     200

601  cctatagtgcgtaaagttgacaaagacaaggtccaacacggcgtcaaagttgttgctctt   660
201  P  I  V  R  K  V  D  K  D  K  V  Q  H  G  V  K  V  V  A  L     220

661  tgtaaagccgaagattctacaactgccgctgaaggcgtccaggaattcgaatgtgacaac   720
221  C  K  A  E  D  S  T  T  A  A  E  G  V  Q  E  F  E  C  D  N     240

721  gggaagtggaagccagagcccgtgccctgcccctaa                           756
241  G  K  W  K  P  E  P  V  P  C  P  -                             251
```

B

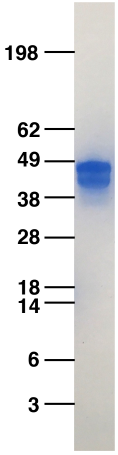

Supplementary Figure 2 (Related to Figure 2):

A. HpARI cDNA sequence showing intron/exon boundaries (green/yellow highlight) and amino acid domains (coloured boxes/text, red = signal peptide, green = CCP1, salmon = CCP2, pink = CCP3)

B. Coomassie-stained purifed HpARI (2 μg), purified by nickel affinity chromatography.

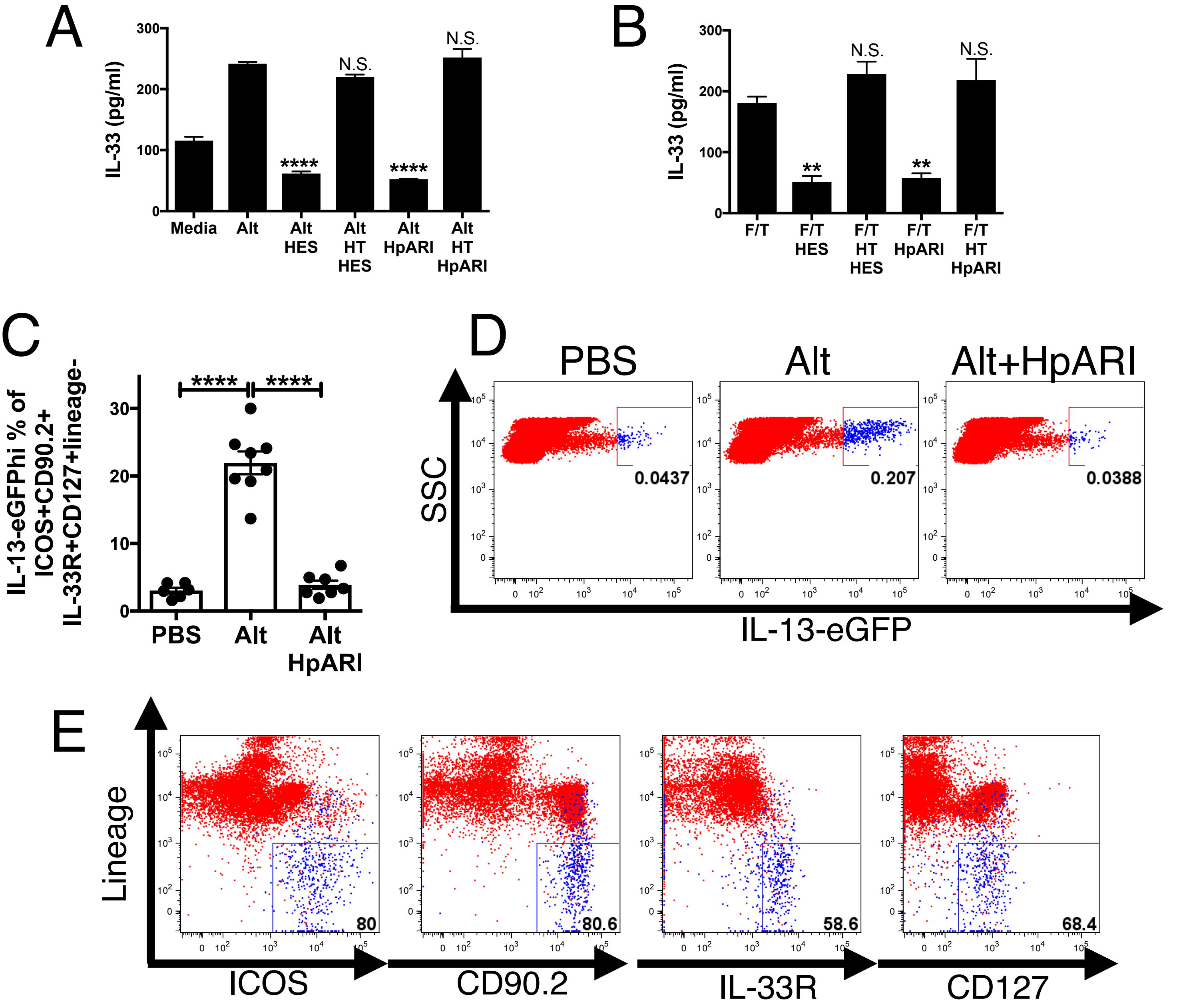

Supplementary Figure 3 (Related to Figure 3):

(A) Naive mouse lung cells were cultured in the presence of Alternaria (200 µg/ml) and 10 µg/ml HES or HpARI for 1 h or heat-treated (95°C for 30 min) HES or HpARI (HT HES or HT HpARI). IL-33 in supernatants was measured by ELISA.

(B) Naive murine lung cells were subjected to freeze/thaw treatment (F/T) in the presence of HES, HpARI, HT HES or HT HpARI. IL-33 in supernatants was measured by ELISA.

**** = p< 0.0001, ** = p<0.01, N.S. = Not significant (p>0.05), compared to Alternaria or freeze/thaw (F/T) control. Standard error of means of 3 replicates, representative of 2-3 repeat experiments.

(C) IL-13-eGFP^{hi} proportion of ICOS+CD90.2+IL-33R+CD127+lineage– ILC2s in the lung, 24 h after intranasal administration of Alternaria allergen and HpARI to IL-13-eGFP reporter mice. Results pooled from 2 repeat experiments. **** = p< 0.0001

(D) Representative FACS plots of IL-13-eGFP in live CD45+ lung lymphocytes from mouse shown in (C)

(E) Representative FACS plots of IL-13-eGFP^{hi} cells (blue) and total CD45+ live lung lymphocytes (red) from an Alternaria-treated IL-13-eGFP reporter mouse.

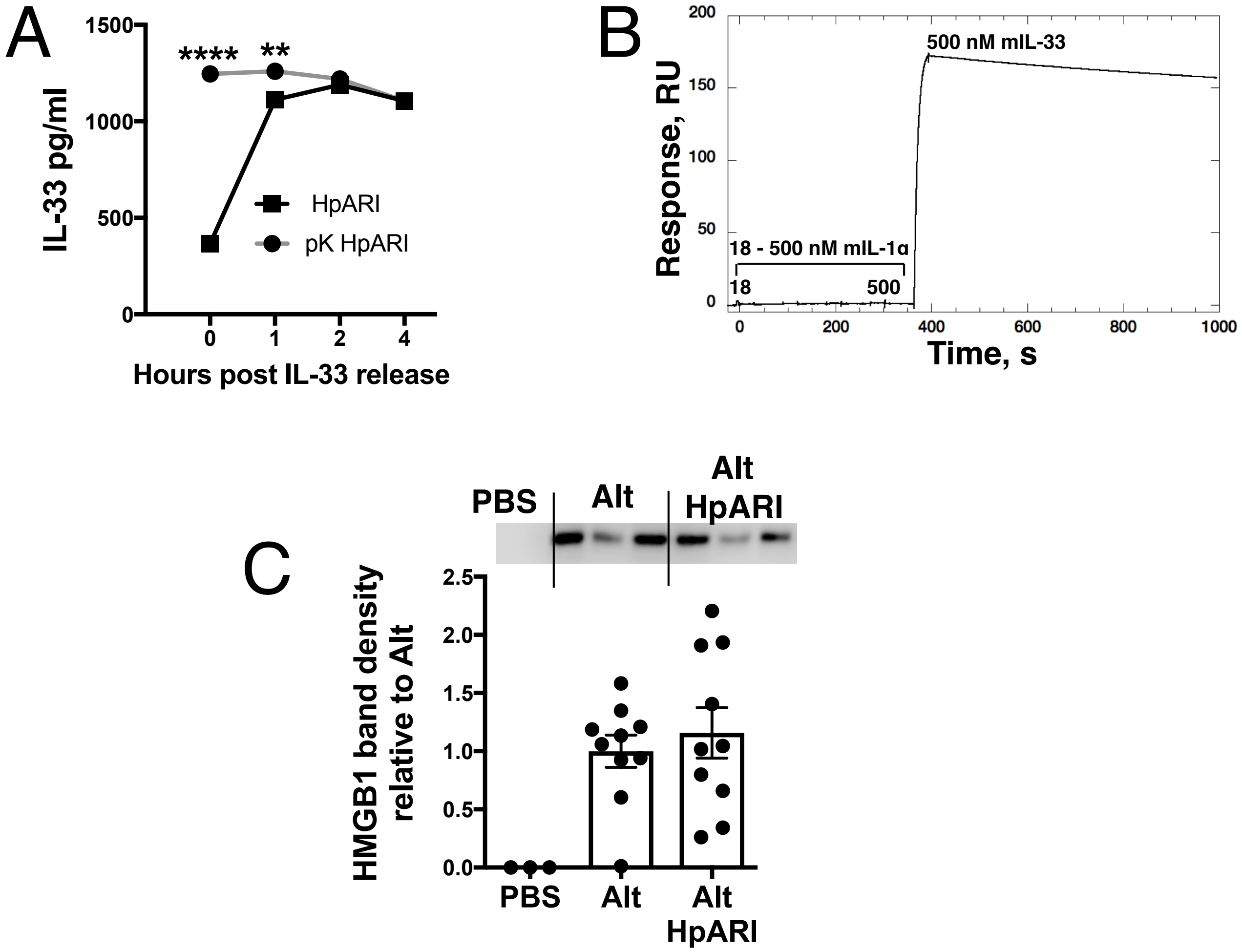

Supplementary Figure 4 (Related to Figure 5):
(A) CMT-64 cell supernatants were taken after freeze-thaw and treated with HpARI or proteinase K-digested and heat-treated HpARI over a timecourse after thaw, as in Figure 5C
(B) Surface plasmon resonance was carried out using mIL-1a or mIL-33 as a ligand for HpARI.
(C) HMGB1 western blots taken 15 min after Alternaria+/- HpARI administration. Pooled from 3 replicate experiments.

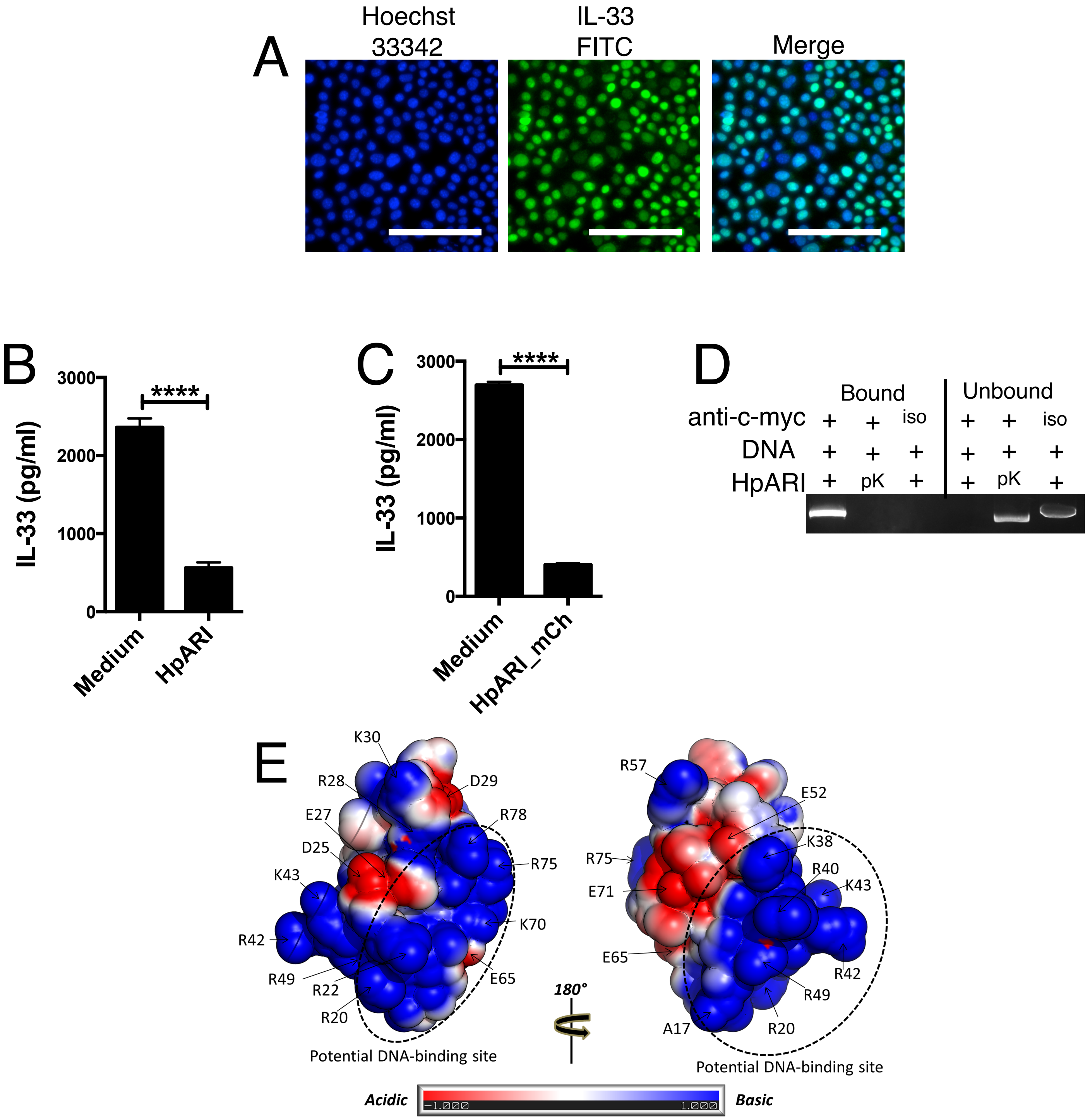

Supplementary Figure 5 (Related to Figure 7):

(A) CMT-64 cells were fixed and permeabilised, before staining with goat anti-mouse IL-33, followed by rabbit anti-goat IgG FITC secondary and counterstained with Hoechst 33342 nuclear stain. Scale bars indicate 100 µm.

(B) CMT-64 cells were subjected to freeze/thaw necrosis in the presence of 5 µg/ml HpARI and IL-33 in the supernatant measured by ELISA.

(C) CMT-64 cells were subjected to freeze/thaw necrosis in the presence of 5 µg/ml HpARI_mCherry and IL-33 in the supernatant measured by ELISA. **** = $p<0.0001$

(D) Immunoprecipitation of plasmid DNA by myc-tagged HpARI, but not by proteinase K and heat treated HpARI (pK) nor with isotype control antibody.

(E) Two views rotated by 180° along the y-axis depicting the electrostatic surface map of the 3-D model of HpARI CCP1 (calculated using APBS) revealing key residues contributing to the highly basic surface (coloured blue) that may confer DNA-binding properties; acidic surface residues are shown in red. A scaled range of -1kT to +1 kT was used, where k=Boltzman's constant and T= Temperature in Kelvins.
